# A pluripotent stem cell-based model for post-implantation human amniotic sac development

**DOI:** 10.1038/s41467-017-00236-w

**Published:** 2017-08-08

**Authors:** Yue Shao, Kenichiro Taniguchi, Ryan F. Townshend, Toshio Miki, Deborah L. Gumucio, Jianping Fu

**Affiliations:** 1 Department of Mechanical Engineering, University of Michigan, Ann Arbor, MI 48109 USA; 2 Department of Cell and Developmental Biology, University of Michigan Medical School, Ann Arbor, MI 48109 USA; 3 Department of Biochemistry and Molecular Biology, Keck School of Medicine, University of Southern California, Los Angeles, CA 90089 USA; 4 Department of Biomedical Engineering, University of Michigan, Ann Arbor, MI 48109 USA

## Abstract

Development of the asymmetric amniotic sac—with the embryonic disc and amniotic ectoderm occupying opposite poles—is a vital milestone during human embryo implantation. Although essential to embryogenesis and pregnancy, amniotic sac development in humans remains poorly understood. Here, we report a human pluripotent stem cell (hPSC)-based model, termed the post-implantation amniotic sac embryoid (PASE), that recapitulates multiple post-implantation embryogenic events centered around amniotic sac development. Without maternal or extraembryonic tissues, the PASE self-organizes into an epithelial cyst with an asymmetric amniotic ectoderm-epiblast pattern that resembles the human amniotic sac. Upon further development, the PASE initiates a process that resembles posterior primitive streak development in a *SNAI1*-dependent manner. Furthermore, we observe asymmetric BMP-SMAD signaling concurrent with PASE development, and establish that BMP-SMAD activation/inhibition modulates stable PASE development. This study reveals a previously unrecognized fate potential of human pluripotent stem cells and provides a platform for advancing human embryology.

## Abstract (web summary)

Early in human embryonic development, it is unclear how amniotic sac formation is regulated. Here, the authors use a human pluripotent stem cell-based model, termed the post-implantation amniotic sac embryoid, to recapitulate early embryogenic events of human amniotic sac development.

## Introduction

**Fig. 1:**
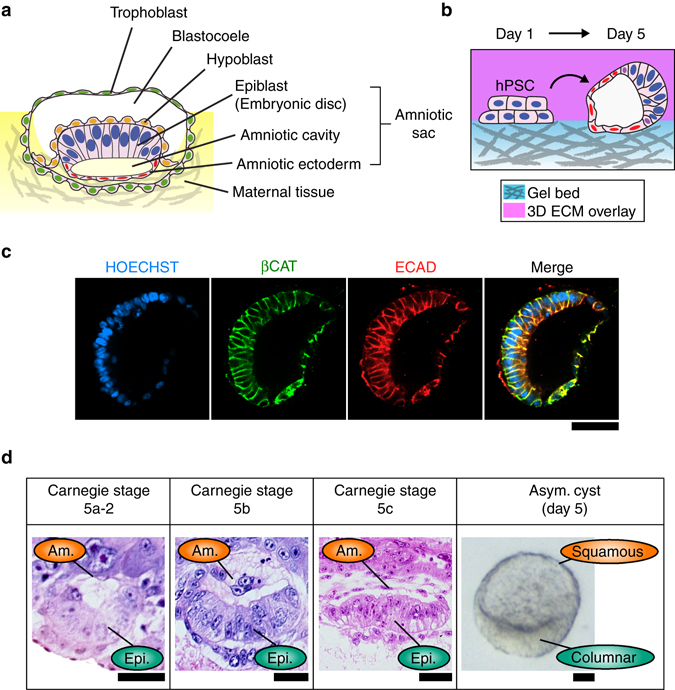
hPSC form asymmetric epithelial cysts in a 3D amniogenic culture system. **a** Cartoon of an implanting human embryo. The amniotic ectoderm, amniotic cavity, and epiblast constitute the amniotic sac. **b** Schematic of the 3D culture system and the formation of asymmetric cysts from hPSC. The culture substrate is made of a thick layer of Geltrex^TM^ (with a thickness >100 µm). The ECM overlay was applied from day 1 onward to provide the 3D culture environment. Asymmetric cysts were consistently observed on day 5 in all (*n* > 18) independent experiments. **c** Representative confocal micrographs showing an asymmetric cyst on day 5, stained for βCAT (*green*) and ECAD (*red*). HOECHST (*blue*) counterstains nuclei. *n* = 3 independent experiments. *Scale bar*, 50 µm. **d** Carnegie stage (CS) 5a-2, 5b, and 5c human embryo sections, showing prospective and definitive amniotic ectoderm (*Am*.) and epiblast (*Epi*.). Phase contrast image shows a representative asymmetric cyst with a distinct bipolar morphological pattern. *Scale bars*, 30 µm

During human embryo implantation, the embryonic inner cell mass gives rise to the amniotic sac—an asymmetrically patterned epithelial cyst that encloses the amniotic cavity with squamous amniotic ectoderm at one pole and columnar epiblast at the other (Fig. 1a)^1^. The development of the amniotic sac is the keystone for early human embryogenesis, as the columnar epiblast and the squamous amniotic ectoderm eventually develop into the embryo proper and the enveloping amniotic membrane, respectively, which together constitute the core of a human embryo. Historically, our understanding of the cellular and molecular processes that underpin peri- and post-implantation human amniotic sac development has been limited by the technical and ethical challenges of harvesting and studying early human embryo specimens. Recently, two groups demonstrated that cultured pre-implantation human blastocysts can continue to develop and form an amniotic cavity in vitro; however, the cultured human embryos did not show amniotic epithelium and no asymmetrically patterned amniotic sac-like structure was observed^2, 3^. Given its central role in post-implantation human embryogenesis, there is a critical need for an in vitro platform to model and study key steps involved in human amniotic sac development.

Human pluripotent stem cells (hPSC), which share molecular similarity with the epiblast in the human embryo^4–6^, have been widely utilized for modeling post-gastrulation human development in vitro^7–10^. Recently, we expanded the application of hPSC to model peri-implantation amniogenesis by using a biomimetic three-dimensional (3D) culture system to induce the development of squamous epithelial cysts that closely resemble human amniotic ectoderm from both morphological and transcriptomic perspectives^11^. Herein, we further report that a subset of cysts in this amniogenic 3D culture system appear to recapitulate the development of the human amniotic sac. Specifically, these asymmetric cystic structures form via an active symmetry-breaking morphogenesis that gives rise to a squamous, amniotic ectoderm-like epithelium at one pole and a columnar, embryonic disc-like epithelium at the other, resembling the human amniotic sac in vivo. We, therefore, name this structure post-implantation amniotic sac embryoid (PASE). Strikingly, upon further development, the PASE exhibits cellular and molecular features that mimic the development of the posterior primitive streak (PS), with a requirement for *SNAI1* to fully deploy epithelial-to-mesenchymal transition and cell dissemination. Furthermore, our data reveal that PASE development is accompanied by an asymmetric pattern of BMP-SMAD signal transduction and that balanced activation/inhibition of the BMP-SMAD signaling pathway modulates stable PASE development. Together, this study presents the first hPSC-based system that models multiple events of human post-implantation amniotic sac development, thereby providing a platform for advancing our fundamental understanding of the morphogenesis, cell fate-patterning, and developmental regulations that characterize early human embryogenesis.

## Results

### hPSC form asymmetric cysts in an amniogenic system

We recently developed a biomimetic 3D culture system to induce the development of human amniotic ectoderm-like tissue from hPSC in vitro^11^. In this system, hPSC are plated as single cells at 30,000 cells cm^−2^ onto a thick, soft gel bed of Geltrex^TM^, in mTeSR1 medium supplemented with 4% (*v*/*v*) Geltrex^TM^ to establish a 3D extracellular environment (see Methods). Intriguingly, in addition to the uniformly squamous amniotic ectoderm-like cysts previously reported in this system^11^, we consistently observed a small population of asymmetric cysts on day 5 in culture (Fig. 1b; Supplementary Fig. 1), suggesting concomitant development of a more complex structure.

These asymmetric cysts are E-CADHERIN+/β-CATENIN + (ECAD+/βCAT+) epithelial sacs composed of flattened, squamous cells on one side, and tall, columnar cells on the other (Fig. 1c). Morphologically, they resemble the bipolar amniotic ectoderm-epiblast pattern seen in the human amniotic sac at Carnegie stages 5a-2, 5b, and 5c, on day past fertilization (d.p.f.) 8, 9, and 12, respectively (Fig. 1d). Specifically, the squamous-columnar morphological transition in the asymmetric cysts mirrors the amniotic ectoderm-epiblast partition in the human amniotic sac, as revealed by quantitated region-specific nuclear dimension and epithelial thickness (Supplementary Fig. 2). These asymmetric cysts are also apico-basally polarized with EZRIN+, WGA-enriched apical surfaces facing inward (Supplementary Fig. 3). Notably, asymmetric cysts frequently form with the squamous side oriented toward the underlying gel bed (Supplementary Fig. 4).

**Fig. 2:**
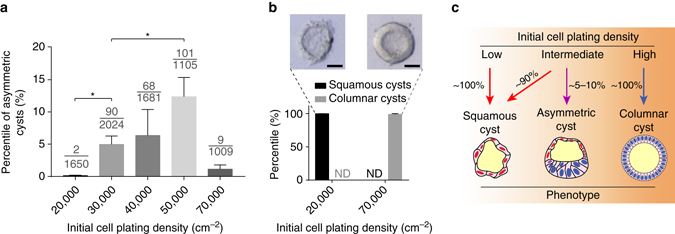
Asymmetric cyst formation is sensitive to initial cell plating density. **a** *Bar plot* showing the percentage of asymmetric cysts formed on day 4 at different initial plating densities (indicated on the *x*-axis); all other conditions are identical. Data represent the mean ± s.e.m. The denominator of each fraction indicates the total number of cysts quantitated for that condition; the numerator of each fraction indicates the number of asymmetric cysts among the quantitated cysts. *P*-values were calculated using unpaired, two-sided Student’s *t*-test. *P < 0.05. *n* = 3–5 biological replicates. *n* = 3 independent experiments. **b** *Bar plot* showing the percentage of fully squamous amniotic ectoderm-like cysts and fully columnar cysts under low-extreme and high-extreme cell plating densities. *ND* not detected. Phase-contrast images show representative squamous (*left*) and columnar (*right*) tissue phenotypes observed at these extreme conditions. *n* = 3 independent experiments. *Scale bars*, 30 µm. **c** Schematic summarizing the effect of initial cell plating density on the formation of the asymmetrically patterned epithelial cysts

Given recent reports regarding the important roles of initial culture parameters such as cell plating density and cell cluster or colony size in pluripotent stem cell (PSC)-based developmental models^7, 12–14^, we specifically examined whether cell plating density affects the asymmetric cyst formation. Indeed, our data reveal a clear dependence on initial cell plating density, with an optimal density in the intermediate range (30,000–50,000 cells cm^−2^) (Fig. 2a). Interestingly, at the highest plating density tested (70,000 cells cm^−2^), cysts are exclusively columnar in culture on day 4 (Fig. 2b). In contrast, at the lowest plating density tested (20,000 cells cm^−2^), the entire culture contains squamous amniotic ectoderm-like cysts on day 4 (Fig. 2b). Together, these data suggest that initial cell plating density modulates the formation of asymmetric cysts in this 3D amniogenic system (Fig. 2c). In this study, an intermediate cell plating density (30,000–35,000 cells cm^−2^) was selected as a default culture parameter unless otherwise noted.

### Asymmetric cysts resemble the post-implantation amniotic sac

**Fig. 3:**
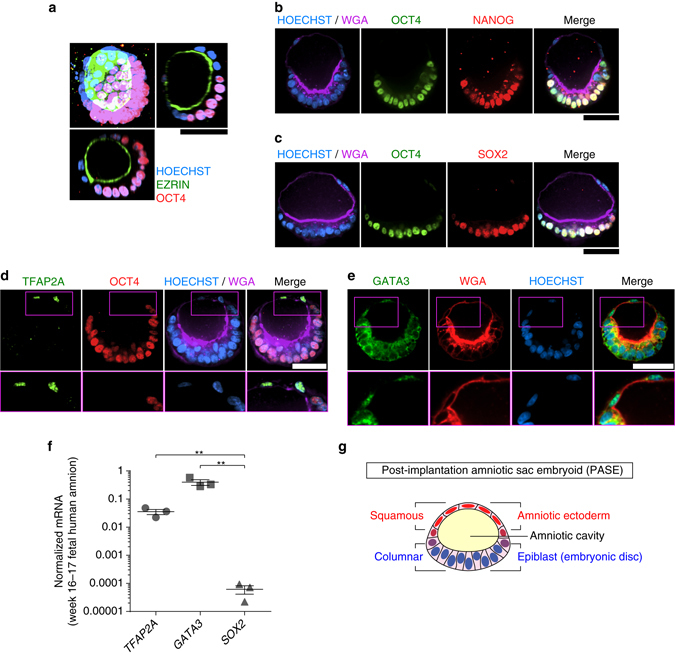
Asymmetric cyst resembles the post-implantation human amniotic sac. **a** 3D rendering of a representative asymmetric cyst on day 5, stained for EZRIN (*green*) and OCT4 (*red*), flanked by *X-Z* (*bottom*) and *Y-Z* (*right*) views. HOECHST (*blue*) counterstains nuclei. *n* = 4 independent experiments. *Scale bar*, 50 µm. **b** Representative confocal micrographs showing a day 5 asymmetric cyst stained for OCT4 (*green*), NANOG (*red*), and WGA (wheat germ agglutinin; *purple*). HOECHST (*blue*) counterstains nuclei. *n* = 6 independent experiments. *Scale bar*, 50 µm. **c** Representative confocal micrographs showing a day 5 asymmetric cyst stained for OCT4 (*green*), SOX2 (*red*), and WGA (*purple*). HOECHST (*blue*) counterstains nuclei. *n* = 3 independent experiments. *Scale bars*, 50 µm. **d** Representative confocal micrographs showing a day 5 asymmetric cyst, stained for TFAP2A (*green*), OCT4 (*red*), and WGA (*purple*). HOECHST (*blue*) counterstains nuclei. Zoom-in images are shown for the *boxed* regions at the amniotic pole. *n* = 3 independent experiments. *Scale bar*, 50 µm. **e** Representative confocal micrographs showing a day 5 asymmetric cyst stained for GATA3 (*green*) and WGA (*red*). HOECHST (*blue*) counterstains nuclei. Zoom-in images are shown for the *boxed* regions at the amniotic pole. *n* = 2 independent experiments. *Scale bar*, 50 µm. **f** qRT-PCR analysis of *TFAP2A*, *GATA3*, and *SOX2* in week 16–17 human fetal amniotic epithelial cells. Data were normalized against *GAPDH* and plotted as the mean ± s.e.m. with *n* = 3 biological replicates. *P*-values were calculated using paired, two-sided Student’s *t*-test. **P < 0.01. **g** Schematic of the asymmetric cyst. The squamous side resembles the amniotic ectoderm lining the roof of the amniotic sac, while the columnar side recapitulates the embryonic disc lining the floor of the amniotic sac, with the amniotic cavity enclosed within. Together, the asymmetric cyst models the morphology and cell fate pattern of the amniotic sac in the post-implantation human embryo. The asymmetric cyst is thus termed the post-implantation amniotic sac embryoid (*PASE*)

We next sought to characterize the cell fates within the asymmetric cyst using immunofluorescence analysis. The results reveal that the columnar side of the asymmetric cyst is composed of cells that prominently retain the pluripotency marker OCT4 (also known as POU5F1), which is lost in the squamous cells (Fig. 3a; Supplementary Fig. 5). Co-staining of OCT4 with other pluripotency markers—NANOG and SOX2—confirms that the columnar side of the asymmetric cyst is composed of undifferentiated, epiblast-like cells (Fig. 3b, c), resembling the embryonic disc at one pole of the human amniotic sac (Fig. 1a). Consistent with this contention, OCT4/NANOG co-staining as well as SOX2 staining have been seen exclusively in the embryonic disc of post-implantation cynomolgus monkey embryos in recent publications^6, 15^. Notably, asymmetrically patterned cysts with these characteristics were consistently generated from three human embryonic stem cell (hESC) lines (H7, H9, and UM63-1), as well as a human-induced pluripotent stem cell (hiPSC) line 1196a (Supplementary Fig. 6).

The squamous side of the asymmetric cyst, in contrast, is composed of a flattened, differentiated epithelium that we recently identified as early human amniotic ectoderm-like tissue^11^. Indeed, TFAP2A and GATA3—two markers for hPSC-derived early human amniotic ectoderm-like tissue^11^—are expressed exclusively in these squamous cells (Fig. 3d, e; Supplementary Fig. 7). Quantitative reverse transcription PCR (qRT-PCR) analysis shows high mRNA levels for *TFAP2A* and *GATA3* in week 16–17 human fetal amniotic epithelium, further supporting the contention that TFAP2A+/GATA3 + squamous cells molecularly resemble human amniotic cells (Fig. 3f). Together, these results show that hPSC can spontaneously self-organize into asymmetric epithelial cysts that resemble the human amniotic sac at post-implantation stages, featuring a central amniotic cavity that is surrounded by a continuous epithelium with a bipolar amniotic ectoderm-epiblast pattern. Such hPSC-derived asymmetric cysts are thus henceforth termed PASE (Fig. 3g).

### PASE develop via active symmetry breaking

**Fig. 4:**
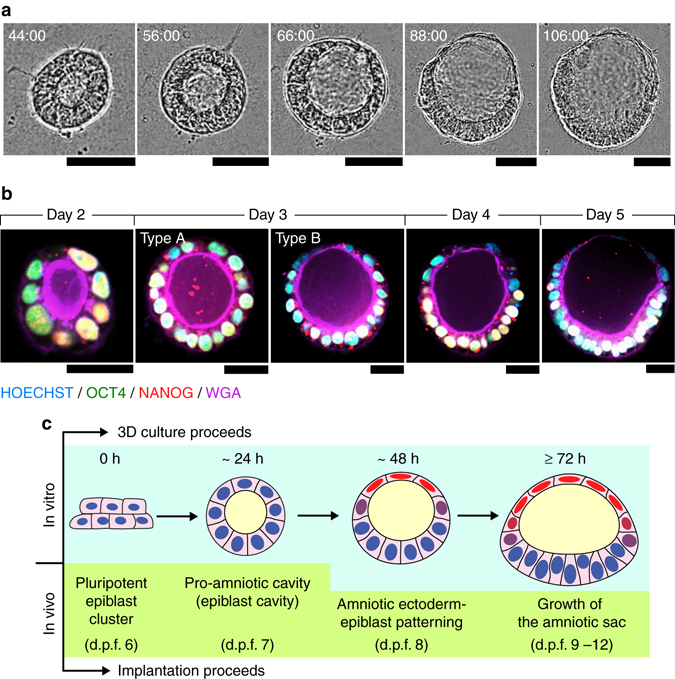
Developmental trajectory of PASE. **a** Representative time-lapse phase-contrast images showing dynamic morphogenesis during the development of a PASE. Time stamps indicate the total hours of culture. *n* = 3 independent experiments. *Scale bars*, 50 µm. Also see Supplementary Movie 1. **b** Confocal micrographs showing representative PASE on day 2, 3, 4, and 5, respectively, stained for OCT4 (*green*), NANOG (*red*), and WGA (*purple*). HOECHST (*blue*) counterstains nuclei. See Supplementary Fig. 8a for images from separate channels. *n* = 3 independent experiments. Early stage (day 2) PASE displayed an asymmetrically positioned lumenal cavity within a cyst that still exhibits a columnar morphology and is positively stained for both OCT4 and NANOG. *Scale bars*, 30 µm. **c** Cartoon showing the time course of PASE development in vitro, compared with human amniotic sac development in vivo

We next examined the morphogenic process of PASE formation using live-cell imaging. Strikingly, our data show that following the formation of a columnar cyst containing a central lumenal cavity, the PASE continues to develop through an active symmetry-breaking process that establishes the bipolar amniotic ectoderm-epiblast pattern, with the cyst wall continuously thinning only at the prospective amniotic pole (Fig. 4a; Supplementary Movie 1). The time course of PASE development was further examined by immunofluorescence analysis. On day 2, most hPSC form lumenal cysts that express both OCT4 and NANOG (Fig. 4b; Supplementary Fig. 8a). At this point, some cysts exhibit an eccentrically positioned lumenal cavity; this represents the earliest stage of PASE development and resembles the Carnegie stage 5a-1 (d.p.f. 7) embryo, featuring a pro-amniotic cavity surrounded by polarized epiblast cells (Supplementary Fig. 8b). On day 3, two types of PASE are observed: one (type A, 20/46) exhibits slight loss of NANOG, but not OCT4, in slightly flattened cells at one pole, while the other (type B, 26/46) shows more markedly flattened cells at the presumptive amniotic pole, with loss of both NANOG and OCT4, representing a further developed stage (Fig. 4b; Supplementary Fig. 8a). Thus, PASE development on day 3 exhibits prominent asymmetry in both morphology and molecular features, with establishment of an amniotic ectoderm-epiblast pattern in vitro that appears to recapitulate the Carnegie stage 5a-2 (d.p.f. 8) embryo (Supplementary Fig. 8b). From day 4–5, PASE maintain the amniotic ectoderm-epiblast pattern (Fig. 4b; Supplementary Fig. 8a) and resemble the growing amniotic sac from Carnegie stage 5a-2 to 5b (d.p.f. 9) and 5c (d.p.f. 12) (Supplementary Fig. 8b). Altogether, PASE development in vitro recapitulates the progressive, asymmetric morphogenesis, and cell fate patterning observed during peri- and post-implantation human amniotic sac development (Fig. 4c). Importantly, a recent study of developing cynomolgus monkey embryos in vivo from E11 to E15 reported a morphological and cell fate pattern in the monkey amniotic sac that parallels our observations herein with PASE development in vitro^15^.

### PASE evolve with posterior PS-like development

**Fig. 5:**
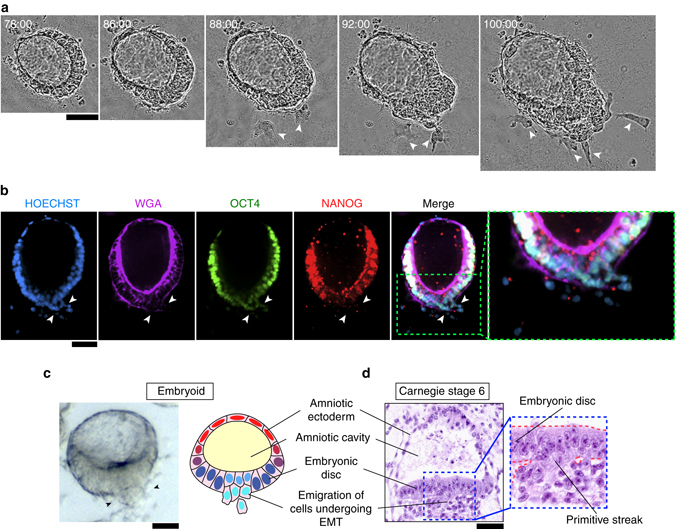
PASE exhibit a phenotype resembling primitive streak development. **a** Representative time-lapse phase-contrast images of a PASE showing progressive emergence of the epithelial-to-mesenchymal (*EMT*) and primitive streak (PS)-like phenotype. *White arrowheads* mark single cells emigrating from the columnar embryonic disc. Time stamps indicate the total hours of culture. *n* = 3 independent experiments. *Scale bar*, 50 µm. Also see Supplementary Movie 2. **b** Representative confocal micrographs showing cells emigrating from the embryonic disc of a PASE on day 5. The PASE was stained for WGA (*purple*), OCT4 (*green*), and NANOG (*red*). HOECHST (*blue*) counterstains nuclei. *n* = 6 independent experiments. *Scale bar*, 50 µm. **c** Phase contrast image (*left*) showing a representative PASE exhibiting cell dissemination (marked by *arrowheads*) from the columnar embryonic disc. Cartoon (*right*) summarizing the anatomy of a PASE with the PS-like phenotype. PASE with PS-like phenotypes were consistently observed on day 5 in all *n* > 18 independent experiments. *Scale bar*, 50 µm. **d** Carnegie stage 6 human embryo section showing the PS. *Scale bar*, 50 µm

Interestingly, our live-cell imaging data reveal that some PASE develop further to exhibit an additional phenotype, with cells focally emigrating from, and only from, the embryonic disc lining the columnar pole (Fig. 5a; Supplementary Movie 2). Immunofluorescence analysis further demonstrates that on day 5, in some PASE (96/304), epithelial structure and OCT4/NANOG expression are disrupted around the cell egression site (Fig. 5b). In these PASE, SOX2—a marker for pluripotent epiblast cells as well as neuroectodermal cells^16^—is also notably downregulated near the cell dissemination region, consistent with exit from pluripotency and suggesting a non-neuroectodermal differentiation of these cells (Supplementary Fig. 9). Morphologically, these locally emigrating cells appear to be undergoing an epithelial-to-mesenchymal transition (EMT), a phenotypic feature associated with PS initiation that can be seen in the Carnegie stage 6 embryo^17^ (Fig. 5c, d).

**Fig. 6:**
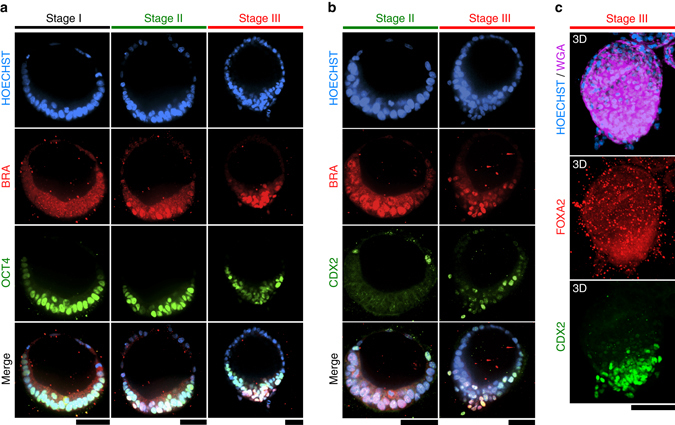
PASE exhibit posterior PS-like molecular characteristics. **a** Representative confocal micrographs showing PASE with different spatial patterns of BRACHYURY (BRA; *red*) and OCT4 (*green*) on day 5. HOECHST (*blue*) counterstains nuclei. *n* = 4 independent experiments. *Scale bars*, 50 µm. **b** Representative confocal micrographs showing PASE with different spatial patterns of BRA (*red*) and CDX2 (*green*) on day 5. HOECHST (*blue*) counterstains nuclei. *n* = 3 independent experiments. *Scale bars*, 50 µm. The differential patterns shown in **a**, **b** reveal three distinct in vitro stages, which are named stages I, II, and III, respectively. **c** 3D rendering of a representative stage III PASE, stained for WGA (*purple*), FOXA2 (*red*), and CDX2 (*green*). HOECHST (*blue*) counterstains nuclei. No nuclear FOXA2 staining was observed in stage III PASE. *n* = 2 independent experiments. *Scale bar*, 100 µm

To molecularly assess the development of PASE with a PS-like phenotype, we examined the expression of BRACHYURY (BRA), a transcription factor associated with PS development^18^, in day 5 PASE. We identified three distinct patterns of BRA expression, based on which we define three consecutive stages of PASE development (Fig. 6a). Stage I (59/173) includes PASE that exhibit no cell dissemination and no prominent nuclear BRA in the embryonic disc. Stage II (56/173) defines PASE that express nuclear BRA in the embryonic disc, but without cell emigration. Stage III (58/173) describes PASE with cells emigrating from a BRA+, PS-like region flanked by the BRA− columnar embryonic disc (Fig. 6a). Immunofluorescence analysis of OCT4 confirms a stage-wise loss of pluripotency that parallels the formation of the BRA+, PS-like region (Fig. 6a). Such stage-dependent modulation of OCT4 and BRA, and the correlated phenotypic evolvement of PASE, show a resemblance to PS development in post-implantation monkey embryos reported in recent publications^6, 15^.

Additional molecular markers were used to further assess the identity of the PS-like developmental structure observed in PASE. CDX2—a marker for posterior/late PS and mesoderm^19^ (Supplementary Fig. 10a)—is expressed in the PS-like region in stage III, but not stage II, PASE (Fig. 6b). Such CDX2 expression in emigrating cells is also consistent with the single-cell transcriptome reported recently for gastrulating cells in monkey embryos^6^. Instead, for stage II PASE, CDX2 is only expressed at the amniotic pole (Fig. 6b), consistent with our recent finding of CDX2 as an early human amniotic marker^11^. In addition, MSX1, which has been found at the posterior end of PS in early mouse embryos^20^, was prominently expressed in emigrating cells of stage III PASE (Supplementary Fig. 10b, c), confirming that these cells resemble posterior PS/mesoderm cells. Of note, MSX1 is also expressed at the amniotic pole (Supplementary Fig. 10b), consistent with previous findings showing MSX1 expression in mouse amniotic cells^20, 21^. In contrast, FOXA2—a marker for anterior PS/endoderm^19, 22^ (Supplementary Fig. 10d)—was absent in PASE (Fig. 6c). SOX17—a marker for endoderm and primordial germ cells (PGCs)^15^—was absent in the PASE as well (Supplementary Fig. 10e). It is also of note that distinct PS elongation as in the early embryo was not observed in the PASE.

**Fig. 7:**
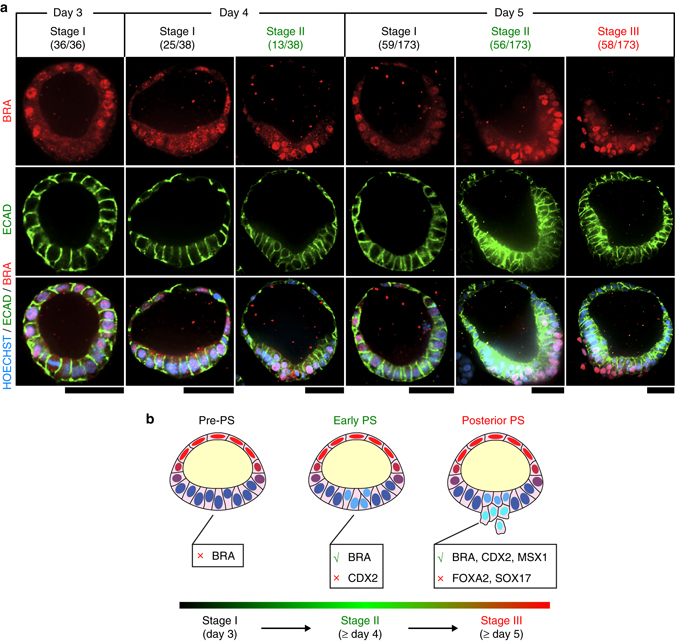
PASE model progressive stages of posterior PS development. **a** Representative confocal micrographs showing time-dependent, stage-wise spatial patterns of BRA (*red*) and ECAD (*green*) in PASE on day 3, 4, and 5, respectively. HOECHST (*blue*) counterstains nuclei. *n* = 2 independent experiments. Stage I (pre-PS), stage II (early PS), and stage III (posterior PS) PASE were observed to emerge sequentially in culture. The denominator reflects the total number of PASE quantitated for that day, while the numerator is the number of PASE at each stage among the quantitated PASE for that day. PASE were distinguished from squamous cysts and selected for quantitation based on their distinct asymmetric epithelial morphology. *Scale bars*, 50 µm. **b** Cartoon summarizing the three sequential stages of PASE development, with stage I, II, and III showing molecular features that resemble progressive posterior PS development through the pre-PS, early PS, and posterior PS stages

We next traced dynamic BRA expression during PASE development. On day 3, only stage I PASE are observed, with nuclear BRA evident only at the flattened amniotic side (Fig. 7a), consistent with both our recent study identifying BRA as an early human amniotic ectoderm marker^11^ and another recent study reporting BRA expression in nascent monkey amnion^15^. On day 4, nuclear BRA emerges in the embryonic disc in some, but not all (13/38), PASE, suggesting asynchronous advance to stage II (Fig. 7a). Stage III PASE with single BRA + emigrating cells are evident after day 4 and present in greater proportion on day 5 (58/173) (Fig. 7a). ECAD is concurrently lost in the BRA+, cell-egression region of stages II and III PASE (Fig. 7a), consistent with a canonical EMT process during PS-like development. Together, these results suggest that PASE can develop to model a progressive, posterior-biased PS development in vitro: while stage I PASE recapitulate the post-implantation, pre-PS amniotic sac, stage II PASE mimic early PS initiation and stage III PASE resemble posterior PS patterning (Fig. 7b).

### PASE EMT phenotype requires *SNAI1*

**Fig. 8:**
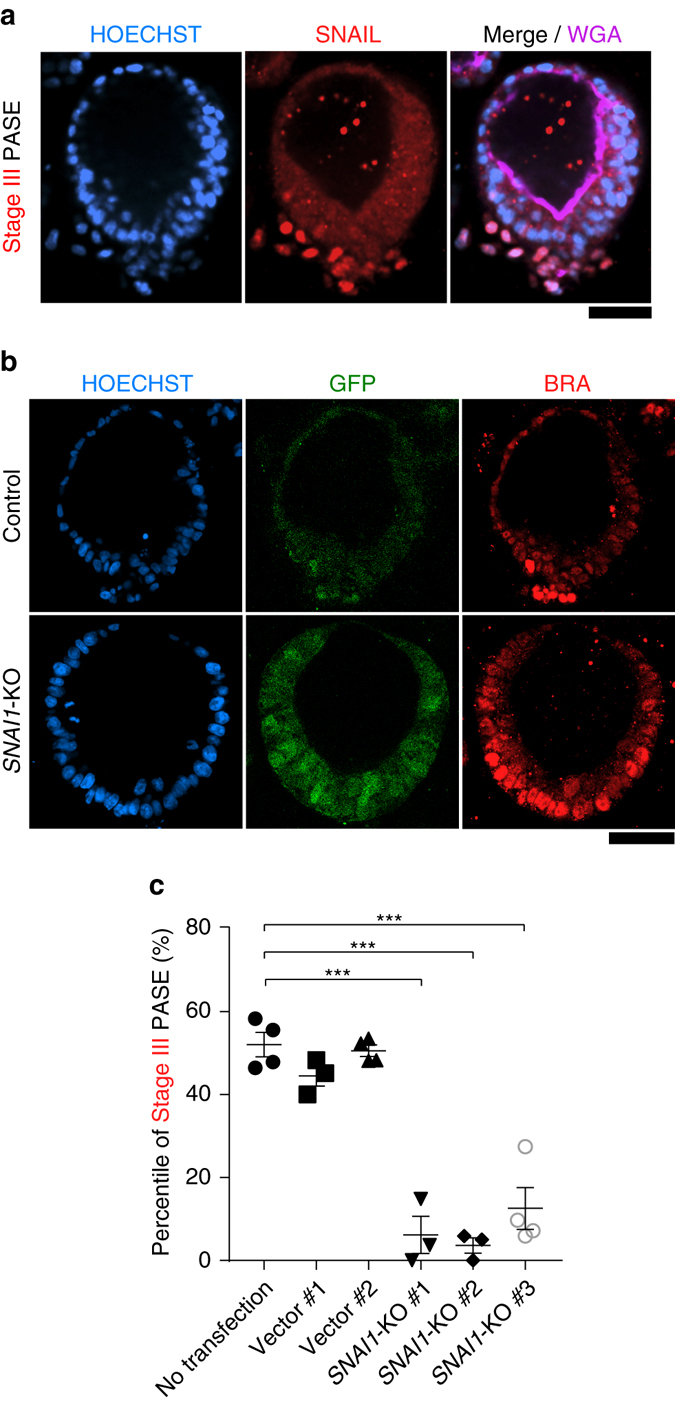
PASE EMT phenotype requires *SNAI1*. **a** Representative confocal micrographs showing a stage III PASE stained for SNAIL (*red*) and WGA (*purple*). HOECHST (*blue*) counterstains nuclei. *n* = 2 independent experiments. *Scale bars*, 50 µm. **b** Confocal micrographs showing GFP expression and BRA staining in a representative PASE generated from control (wild type) cells (*upper*; transfected with piggyBac-SpCas9-T2A-GFP vector only) and *SNAI1*-KO cells (*lower*; transfected with piggyBac-SpCas9-T2A-GFP-h*SNAI1*). HOECHST counterstains nuclei. *Scale bar*, 50 µm. **c** Quantitated percentages of stage III PASE among all PASE when using an untransfected H9 line, two separate H9 lines transfected with piggyBac-SpCas9-T2A-GFP vector only (Control #1 and #2), and three separate *SNAI1*-KO lines, respectively. Data represent the mean ± s.e.m, with *n* = 3–4 biological replicates indicated by individual dots under each condition. *n* = 3 independent experiments. *P*-values were calculated using unpaired, two-sided Student’s *t*-test. ***P < 0.001

Consistent with the above findings, SNAIL—another transcription factor associated with EMT and PS development—was also observed in the posterior PS-like region of stage III PASE (Fig. 8a). Given the importance of SNAIL/*SNAI1* for EMT in gastrulation, as demonstrated in mice^23^, we next examined whether *SNAI1* is a critical mediator of the EMT phenotype observed in stage III PASE. To this end, we generated *SNAI1* knockout (*SNAI1*-KO) hESC (H9) lines using a customized piggyBac transposon vector, integrated with a CRISPR/Cas9 genome editing system containing a guide RNA sequence to target human *SNAI1*: piggyBac-SpCas9-T2A-GFP-h*SNAI1* (Supplementary Fig. 11; see Methods). Control cells were transfected with the piggyBac-SpCas9-T2A-GFP vector lacking the guide RNA. Notably, loss of *SNAI1* significantly suppresses the EMT phenotype and thus stage III PASE (Fig. 8b, c). Interestingly, BRA expression in stage II PASE is not affected by *SNAI1*-KO (Fig. 8b), consistent with previous findings that loss of *Snai1* does not abolish BRA expression during PS development in mice^23^. Together, these data demonstrate that the EMT phenotype of stage III PASE develops in a *SNAI1*-dependent manner.

### BMP-SMAD signaling regulates PASE formation

**Fig. 9:**
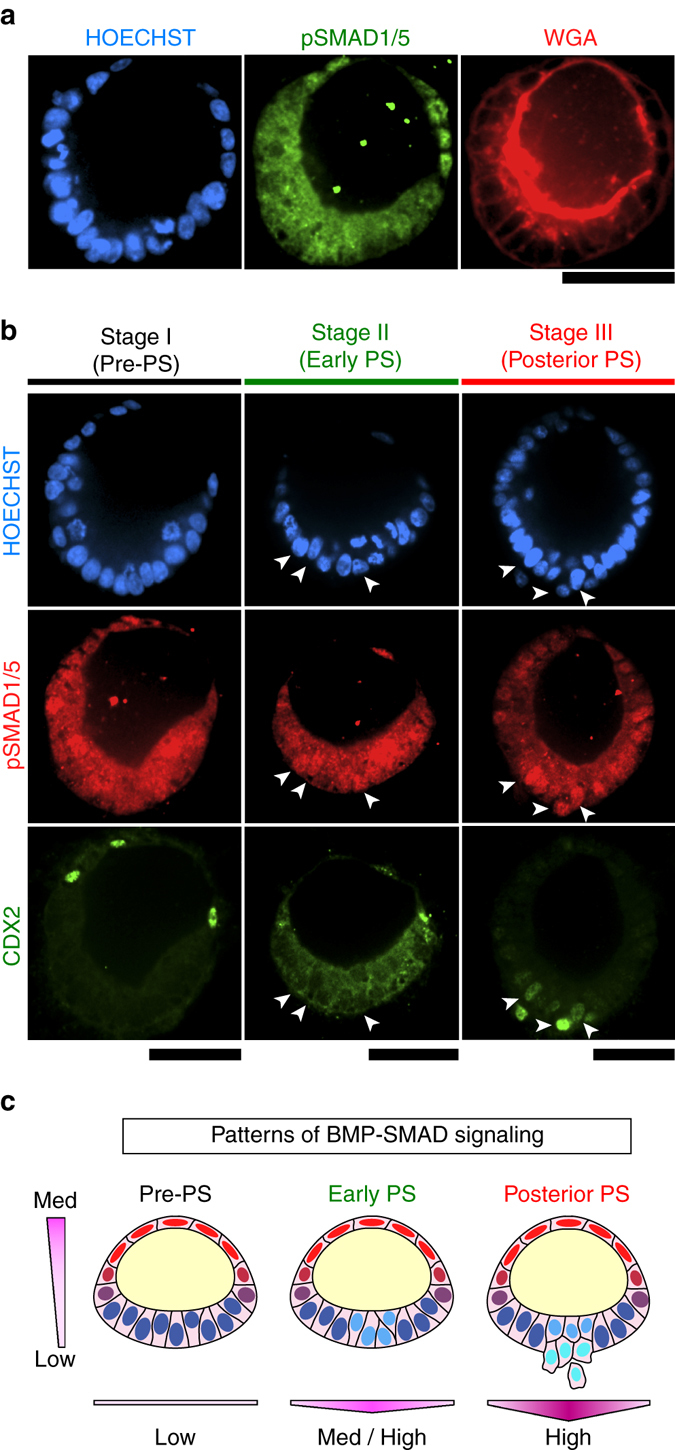
Self-patterned BMP-SMAD signal transduction during PASE development. **a** Representative confocal micrographs showing a pre-PS PASE on day 5 stained for pSMAD1/5 (*green*) and WGA (*red*). HOECHST (*blue*) counterstains nuclei. *n* = 2 independent experiments. *Scale bar*, 50 µm. **b** Representative confocal micrographs showing stage-wise spatial patterns of pSMAD1/5 (*red*) and CDX2 (*green*) in PASE on day 5. HOECHST (*blue*) counterstains nuclei. *Arrow-heads* mark pSMAD1/5 + cells in the embryonic disc of the PASE. *n* = 2 independent experiments. *Scale bars*, 50 µm. **c** Cartoon summarizing spatial patterns of BMP-SMAD signaling in the PASE along the amniotic ectoderm-epiblast axis as well as along the medial-lateral axis of the embryonic disc. *Color scale* represents the intensity of BMP-SMAD signaling ranging from low to medium and high as indicated

During early embryogenesis in mice, BMP-SMAD signaling plays a pivotal role in amniotic tissue specification and morphogenesis, as loss of *Bmp2* or *Smad5* results in defects in both amniotic and embryonic patterning^24, 25^. Interestingly, our previous work showed spontaneous induction of *BMP2/4/7* expression and endogenously activated BMP-SMAD signaling during the development of squamous, human amniotic ectoderm-like cysts from hPSC^11^. Therefore, we next examined BMP-SMAD signaling during PASE development. Immunofluorescence analysis of phosphorylated SMAD1/5 (pSMAD1/5)—a downstream target and activator of BMP-SMAD signaling—reveals a distinct asymmetric pattern of BMP-SMAD signaling, with prominent nuclear pSMAD1/5 exclusively at the amniotic pole of pre-gastrulation (stage I) PASE (Fig. 9a; Supplementary Fig. 12). Stage-dependent patterns of nuclear pSMAD1/5 were further observed (Fig. 9b), mirroring the progressive BRA expression pattern during PASE development (Figs. 6a and 7a). Notably, nuclear pSMAD1/5 emerges in the embryonic disc prior to CDX2 (Fig. 9b), consistent with the recent finding that the activation of BMP-SMAD signaling precedes CDX2-mediated posterior PS specification^22^. Together, these results provide evidence for endogenous, stage-dependent patterns of BMP-SMAD signaling during PASE development in vitro (Fig. 9c).

**Fig. 10:**
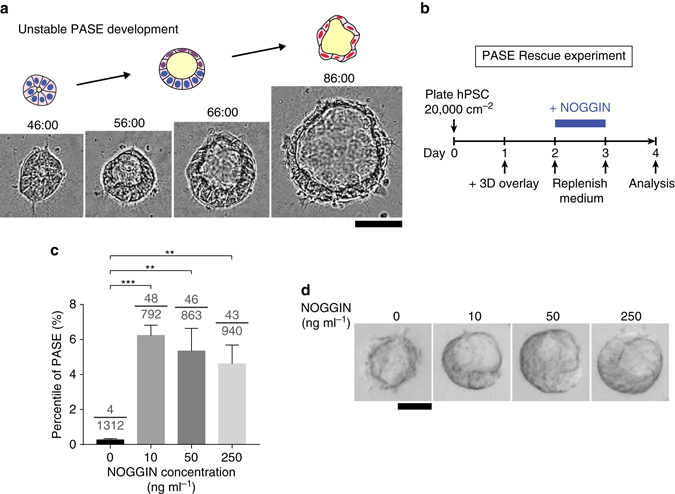
BMP-SMAD activation/inhibition balance modulates PASE formation and stability. **a** Representative time-lapse sequence showing the initial development of an asymmetric cyst and its subsequent conversion into a fully squamous cyst. Time stamps indicate the total hours of culture. *n* = 3 independent experiments. *Scale bar*, 50 µm. Also see Supplementary Movie 3. **b** Schematic outlining the PASE rescue experiment. **c** *Bar plot* showing the percentage of PASE as a function of NOGGIN dosage. Data represent the mean ± s.e.m. The denominator of each fraction indicates the total number of cysts quantitated under that condition; the numerator of each fraction indicates the number of PASE among the quantitated cysts. *P*-values were calculated using unpaired, two-sided Student’s *t*-test. **P < 0.01, ***P < 0.001. *n* = 4 biological replicates. **d** Representative phase-contrast images of squamous amniotic ectoderm-like cysts and asymmetric PASE formed under different NOGGIN dosages as indicated. *n* = 3 independent experiments. *Scale bar*, 50 µm

The data above reveal a correlation between the asymmetric pattern of BMP-SMAD signaling and the symmetry-breaking process during PASE formation, implying that a balance of activation and inhibition of BMP-SMAD signaling might be involved in maintaining the stable amniotic ectoderm-epiblast pattern in PASE. Indeed, further analysis of our live-cell imaging data demonstrates that while PASE initiate from a columnar morphology and continuously develop and mature toward a stable bipolar pattern (Fig. 3a), some cysts (12/150)—herein referred to as unstable PASE—firstly attain a bipolar pattern which then destabilizes as the columnar pole differentiates into a fully squamous amniotic ectoderm-like tissue (Fig. 10a; Supplementary Movie 3). The majority of cysts, however, convert from an initially columnar state to a fully squamous, amniotic ectoderm-like state by circumferential, multi-focal progressive squamous morphogenesis without passing through a notable asymmetric intermediate state (Supplementary Fig. 13; Supplementary Movie 4). The observation of unstable PASE development further supports the potential involvement of a dynamic activation/inhibition equilibrium, perhaps mediated by BMP-SMAD signaling, in regulating the stability of PASE development.

To test this conjecture, we examined whether exogenous inhibition of BMP-SMAD signaling via treatment with NOGGIN—a protein antagonist to BMP2/4/7—could rescue stable PASE formation, under low cell plating density conditions that typically result in a full conversion to squamous amniotic ectoderm-like cysts (Fig. 10b). When hPSC are plated at a low density (20,000 cells cm^−2^) in the absence of NOGGIN, squamous amniotic ectoderm-like tissue formation dominates on day 4, with PASE formation strongly suppressed (Fig. 2). In contrast, with NOGGIN supplementation from day 2–3 (Fig. 10b), stable PASE formation is dramatically rescued on day 4 (Fig. 10c, d). Together, these data support the notion that balanced activation and inhibition of BMP-SMAD signaling may play an important role in maintaining the stable asymmetric amniotic ectoderm-epiblast tissue pattern during PASE development.

## Discussion

In this study, we demonstrate that when cultured in a biomimetic 3D system, hPSC can self-organize to model multiple human embryogenic events, including the morphogenesis and cell fate patterning of the amniotic sac as well as posterior PS formation. These findings unveil a previously unrecognized developmental potential of hPSC and present the first embryoid model for studying post-implantation human amniotic sac development, which is drastically different from that in mice^26, 27^. Although it has been proposed that human amniotic sac development involves an intermediate step in which the epiblast cyst is opened to the trophoblast, forming a tropho-epiblastic cavity (d.p.f. 8)^1, 28^, our data suggest otherwise— the human amniotic sac develops as a continuous epithelial cyst that constantly encloses the (pro-)amniotic cavity while it undergoes symmetry-breaking and amniotic ectoderm-epiblast patterning. This finding is also consistent with other histological studies conducted in humans and non-human primates^15, 26, 29^, supporting the conclusion that the amniotic sac in primates develops through self-organized symmetry-breaking morphogenesis from a primitive lumenal epiblastic cyst.

Similar to the lumenal morphogenesis observed here in PASE, two recent studies using in vitro culture techniques have demonstrated the self-organization of an asymmetrically positioned, presumptively pro-amniotic cavity within OCT4 + epiblast cells in attached human blastocysts^2, 3^. However, further development of a bipolar amniotic sac was unclear in those cultured embryos. The PASE model presented here opens unique opportunities to study the cell and molecular mechanisms that govern the formation of the amniotic sac as well as stability of the amniotic ectoderm-epiblast tissue boundary during post-implantation human development.

Our study demonstrates that the self-organization of PASE involves an endogenously activated program of asymmetrically patterned BMP-SMAD signaling; the balance of BMP-SMAD activation and inhibition in turn modulates PASE stability. However, the molecular mechanism(s) underlying the initial asymmetric activation of BMP-SMAD signaling in PASE remains unclear. Our recent RNA sequencing data reveal that *BMP2/4/7* as well as *NOG* (encoding BMP antagonist NOGGIN) are simultaneously and robustly upregulated in the amniotic ectoderm-like squamous tissue^11^, suggesting the amniotic ectoderm as a potential initiator of an activator/inhibitor feedback system to induce biological patterning of the PASE through principles such as those seen in classic developmental patterning systems^30, 31^. Consistent with this notion, a BMP/NOGGIN-based activator/inhibitor system was recently demonstrated responsible for dictating two-dimensional (2D) biological fate patterns in an hPSC-based gastrulation model^14, 32^. Nevertheless, it remains a future goal to examine the action and roles of individual BMP signaling activators and inhibitors in the formation and stabilization of PASE.

Our findings revealed a prominent dependence of the PASE formation on initial cell plating density. Such dependence on cell plating density has also been reported in other PSC-based developmental models^7, 12–14^. However, a general and unifying mechanistic picture depicting the role of cell plating density in diverse biological patterns formed in PSC-based models remains largely elusive. A recent study on an hPSC-based 2D gastrulation model demonstrated a direct connection between cell density and the spatial localization of cell surface receptors for transforming growth factor-β and BMP signaling, wherein high cell density facilitates the seclusion of receptors to cell–cell contact and thus suppresses cellular responsiveness to soluble ligands^14^. This study presents an interesting paradigm that remains to be examined in the context of PASE development and stabilization in a 3D environment.

Recent work by us and others have supported the role of the amniotic ectoderm as a BMP-producing autocrine and paracrine signaling center in early human embryonic development^11, 15^. The PASE model reported here allows further explorations of the role of the amniotic ectoderm in directing the growth and differentiation of the nearby tissues including the embryonic disc and the PGCs. A recent study on post-implantation monkey embryos demonstrated SOX17+/NANOG + PGC as a distinct, BMP-responsive cell population that resides in and disseminates from the BMP-producing amniotic ectoderm^15^. However, SOX17+/NANOG + PGC were not observed in PASE, possibly due to differences between in vivo and in vitro contexts. Future efforts can be directed to identify additional factors that may interact with BMP-SMAD signaling in PASE to dictate lineage bifurcation between the amniotic ectoderm and PGC.

Furthermore, PASE provide a technical foundation to further study the interaction between the amniotic sac and surrounding extraembryonic tissues during post-implantation human embryogenesis. It remains an important future goal to leverage the PASE model to examine the role for individual extraembryonic tissues, e.g., trophoblast and primitive endoderm^15^, in supporting the formation, growth, and patterning of the human amniotic sac.

Together, we report herein the first hPSC-based embryoid model for post-implantation human amniotic sac development. Our findings provide insight into previously inaccessible but critical embryogenic events in post-implantation human development. Going forward, continuous development of the PASE model will provide a synthetic embryological platform to complement scarce in vivo and ex vivo work that uses live human embryos, thereby opening previously undescribed avenues to advance human embryology, embryo toxicology, and reproductive medicine.

## Methods

### Ethics statement

The PASE model generated in this study lacks primitive endoderm and trophoblast, and thus cannot form yolk sac and placenta. In addition, there is no evidence of the presence of anterior PS cells, endoderm cells, or PGCs in this embryoid system. Instead, only posterior PS/mesoderm cells are found (in addition to amniotic ectoderm-like cells and pluripotent epiblast-like cells). Therefore, the PASE model does not have human organismal form or potential. Furthermore, given that amniotic sac development starts on d.p.f. 7 in vivo, and that all experiments were terminated by no later than day 5 in vitro, the culture of PASE in this study terminates before effectively reaching 14 developmental days. All protocols for culturing the PASE were approved by the Human Pluripotent Stem Cell Research Oversight Committee at the University of Michigan.

### Cell lines

hPSC lines H9 (hESC, WA09, P50, WiCell; NIH registration number: 0062), H7 (hESC, WA07, P52, WiCell; NIH registration number: 0061), UM63-1 (hESC, P25, provided by Dr Gary D. Smith at the University of Michigan MStem Cell Laboratories; NIH registration number: 0277), and 1196a (hiPSC, P42, from the University of Michigan Pluripotent Stem Cell Core^33^) were used in this study. All protocols for the use of hPSC lines were approved by the Human Pluripotent Stem Cell Research Oversight Committee at the University of Michigan. All hPSC lines have been authenticated by the original sources as well as by immunostaining for pluripotency markers and successful differentiation to three germ layer cells. All hPSC lines were authenticated as karyotypically normal at the indicated passage number by Cell Line Genetics (Madison, USA). All hPSC lines tested negative for mycoplasma contamination (LookOut Mycoplasma PCR Detection Kit, Sigma-Aldrich).

### Cell culture

hPSC were maintained in a standard feeder-free culture system using mTeSR1 medium (STEMCELL Technologies) and lactate dehydrogenase-elevating virus (LDEV)-free hESC-qualified reduced growth factor basement membrane matrix Geltrex^TM^ (Thermo Fisher Scientific; derived from Engelbreth-Holm-Swarm tumors similarly as Matrigel®) per the manufacturers’ instructions. All hPSC were used before P70.

### Three-dimensional hPSC culture

A recently developed 3D hPSC amniogenesis protocol was used in this study^11^. In brief, cultured hPSC were dissociated with Accutase (Sigma-Aldrich) at 37 °C for 10 min, centrifuged and resuspended in mTeSR1 containing 10 µM Y27632 (Tocris). hPSC were plated as single cells at 30,000–35,000 cells cm^−2^, unless otherwise specified, onto a thick gel bed with nominal thickness ≥ 100 µm. After 24 h (on day 1), culture medium was replenished with fresh mTeSR1 without Y27632, with 4% (*v*: *v*) Geltrex^TM^ supplemented in the medium as well^34^. Thereafter, mTeSR1 medium was replenished daily, and 4% (*v*: *v*) Geltrex^TM^ was supplemented daily till day 5, unless otherwise noted. No pre-specified size effect is involved in this study. No sample was excluded from this work.

In the PASE rescue experiment, human recombinant NOGGIN (R&D Systems; reconstituted in PBS) was added to the culture medium at 0, 10, 50, and 250 ng ml^−1^ from day 2 to day 3. PBS was added to the control group (0 ng ml^−1^).

### Genome editing for *SNAI1* knockout

pSpCas9-2A-GFP (PX458) construct was obtained from the laboratory of Dr Feng Zhang (Addgene#48138)^35^. Human U6 (hU6) promoter/guide RNA (gRNA) and SpCas9-T2A-GFP were PCR amplified (hU6—forward: 5′-GCACTAGTGAGGG CCTATTTCCCATG-3′; reverse: 5′-CGACTAGTAACGGGTACCTCTAGAGC-3′, and SpCas9-T2A-GFP—forward: 5′-GCGCTAGCGCCACCATGGACTATAAG-3′; reverse: 5′-CGGCGGCCGCTTACTTGTACAGCTCGTC-3′). Amplified products were then subcloned into a pPB transposon vector (piggyBac transposon system obtained from Dr Joseph Loturco, Connecticut USA)^36^ at the SpeI site (hU6), and at NheI and NotI sites (SpCas9-T2A-GFP) to generate a piggyBac-CRISPR/Cas9 vector that contains Cas9 and hU6-gRNA expression cassettes flanked by piggyBac transposon terminal repeat recognition elements.

To introduce insertion/deletion (indel) mutations into exon 1 of the human *SNAI1* (h*SNAI1*) locus ATGCCGCGCTCTTTCCTCGTCAGGAAGCCCTCCGACCCCAATCGGAAGCCTAACTACAGCGAGCTGCAGGACTCTAATCCAG) via non-homologous endjoining (NHEJ), a gRNA targeting sequence (GTTAGGCTTCCGATTGGGGTCGG) was designed (using published algorithms, http://crispr.mit.edu). The annealed oligo containing the gRNA sequence (sense: 5′-CACCGGTTAGGCTTCCGATTGGGGT-3′; anti-sense: 5′-AAACACCCCAATCGGAAGCCTAACC-3′) was subcloned into *Bbs*I sites^35^ to generate the piggyBac-CRISPR/Cas9 construct containing the h*SNAI1* gRNA targeting sequence (piggyBac-CRISPR/Cas9-h*SNAI1*). piggyBac-CRISPR/Cas9-h*SNAI1* was then co-transfected with pCAG-PBase (piggyBac transposase obtained from Dr Joseph Loturco)^36^ using GeneJammer (Agilent Technologies) into H9 hESCs that were plated at 50,000 cells cm^−2^ 24 h prior to transfection.

The sub-confluent culture of H9 piggyBac-CRISPR/Cas9-h*SNAI1* cells was sorted for GFP + cells using fluorescence-activated cell sorting (FACS); FACS sorted cells were subsequently cultured at low density (500 cells cm^−2^) for clonal selection. Established colonies were manually picked and expanded for screening indel mutations using PCR amplification of a region spanning the exon 1 of human *SNAI1* gene (forward: 5′-GCGAATTCAGCGAGTGGTTCT TCTGC-3′; reverse: 5′-CGGCGGCCG CACCTGGATTAGAGTCCT GC-3′). Subsequently, the PCR amplified product was subcloned into pPBCAG-GFP^36^ at EcoRI and NotI sites, and was sequenced (5′-TTATGGTA ATCGTGCGAG AG-3′). At least 12 bacterial colonies were sequenced to confirm genotypic clonality. In control cells, a piggyBac-CRISPR/Cas9 vector lacking the gRNA targeting sequence was co-transfected with pCAG-PBase.

### Derivation of PS cells

Anterior, posterior and late PS cells, respectively, were derived from hPSC by adapting previously published protocols^19^. In brief, hPSC were dissociated to single cells before being plated at 25,000 cm^−2^ in Essential 8^TM^ medium (E8; Thermo Fisher Scientific) containing 10 µM Y27632 onto glass coverslips that were thinly coated with 1% Geltrex^TM^ solution. On day 1, the medium was replenished with fresh E8. On day 2, the medium was replaced with anterior PS, posterior PS, and late PS differentiation medium, respectively, with the following composition: (1) anterior PS differentiation medium: Essential 6^TM^ medium (E6; Thermo Fisher Scientific) supplemented with 20 ng ml^−1^ FGF2 (Peprotech), 10 µM LY294002 (Tocris), 25 ng ml^−1^ BMP4 (R&D Systems), and 50 ng ml^−1^ Activin A (R&D Systems); (2) posterior PS differentiation medium: E6 supplemented with 20 ng ml^−1^ FGF2, 10 µM LY294002, and 50 ng ml^−1^ BMP4; (3) late PS differentiation medium: E6 supplemented with 20 ng ml^−1^ FGF2, and 8 µM CHIR99021 (Tocris). All differentiation protocols were carried out for 48 h before downstream analyses.

### Derivation of definitive endoderm cells

SOX17 + definitive endoderm cells were derived from H9 hESC following an established protocol^37^. In brief, a six-well culture plate of hPSC that were 85–90% confluent were dissociated by incubating in 200 µg ml^−1^ Dispase (Thermo Fisher Scientific) at 37 °C for 10 min, followed by three rinses with DMEM/F12 medium. The dissociated hPSC colonies were triturated and plated at a 1:6 ratio in mTeSR1 medium, at about 5000 colony pieces per well, in a 24-well culture plate that was previously coated with 1% Matrigel (Corning) solution. Fresh mTeSR1 medium was replenished daily for 1 or 2 more days till the culture reached 85–90% confluency. Afterwards, definitive endoderm differentiation was induced by applying day 1 differentiation medium (RPMI medium + 2 mM ʟ-glutamine + 100 ng ml Activin A) for 24 h, followed by day 2 differentiation medium (RPMI medium + 2 mM ʟ-glutamine + 100 ng ml Activin A + 0.2% defined fetal bovine serum (dFBS; Hyclone)) for another 24 h, then day 3 differentiation medium (RPMI medium + 2 mM ʟ-glutamine + 100 ng ml Activin A + 2% dFBS) for 24 h. Cells were fixed after 72 h of differentiation before downstream analysis.

### Basement membrane matrix gel beds

The Geltrex^TM^ gel bed was generated based on a “sandwich” set-up developed recently^11, 38^, unless otherwise noted. In brief, a 22 × 22 mm^2^ glass coverslip (“attaching substrate”) was treated with air plasma (Harrick Plasma) for 2 min, before coating with 0.1 mg ml^−1^ poly-(l-lysine) (PLL) solution (Sigma-Aldrich) for 30 min; 1% glutaraldehyde solution (Electron Microscopy Sciences) was then added for another 30 min. To prepare a “releasing substrate”, a pre-cleaned glass slide was treated with air plasma for 2 min before coating with a 0.1 mg ml^−1^ poly-(l-lysine)-graft-poly-(ethylene glycol) (PLL-g-PEG; SuSoS) solution for 1 h. To obtain gel beds with a nominal thickness of 100 µm, 50 µl undiluted Geltrex^TM^ was sandwiched between the attaching and releasing substrate on ice and incubated at 37 °C for 30 min. The gel bed, attached to the attaching substrate, was then peeled off from the releasing substrate, submerged in DMEM/F12 medium (Thermo Fisher Scientific) and incubated at 37 °C overnight before plating cells. To prepare gel beds with a nominal thickness greater than 100 µm, spacers made of polydimethylsiloxane (PDMS; Dow Corning) films were placed between the attaching and releasing substrates when preparing the gel bed sandwich. The PDMS film was generated by spin-coating PDMS prepolymer (prepared as a mixture of PDMS base and curing agent at a 10:1 ratio) onto a petri dish at 500 rpm for 40 s (for generating 150 µm thick PDMS film used in this study). The PDMS film was cured at 70 °C for at least 24 h before use.

For live-cell imaging, a thick matrix gel bed was directly deposited onto the bottom of a six-well tissue culture plate by pipetting 90 µl of undiluted ice-cold Geltrex^TM^ into each (creating an average nominal gel bed thickness of~150 µm). The deposited gel bed was solidified at 37 °C for 30 min before being incubated in warm DMEM/F12 at 37 °C for 24 h before use. Such gel beds were also used in experiments for examining the effect of *SNAI1*-KO. All samples were randomly allocated to different experimental groups. However, no particular randomization method was used in this work.

### Cell fixation and immunocytochemistry

hPSC were fixed in 4% paraformaldehyde (prepared in 1 × PBS) for 30 min, and permeabilized in 0.1% SDS (sodium dodecyl sulfate, dissolved in PBS) solution for another 30 min. Samples were blocked in 2% goat serum solution (Thermo Fisher Scientific) or donkey serum solution (Sigma-Aldrich) at 4 °C for 24 h before incubation with primary antibody solution at 4 °C for another 24 h. Samples were labeled with goat/donkey secondary antibodies (1:500 dilution) at 4 °C for 24 h. HOECHST 33342 (Thermo Fisher Scientific) was used for counterstaining cell nuclei. Alexa-fluor 555 or 647 dye-conjugated wheat germ agglutinin (WGA; Thermo Fisher Scientific) was used as a pan-cell membrane marker. Primary antibodies and their sources and dilutions are listed in Supplementary Table 1.

### Confocal microscopy and image analysis

Images were acquired on an Olympus 1X81 fluorescence microscope equipped with a CSU-X1 spinning-disc unit (YOKOGAWA) or a Nikon-A1 laser scanning confocal microscope (Nikon). Fluorescence images acquired from confocal microscopy were reconstructed in 3D using Imaris8.2 (Bitplane). Measurements of cell nucleus dimensions, cyst epithelial thickness, and cyst orientation angle were performed manually using the Measurement tool in ImageJ (NIH)^39^. When necessary, 3D reconstructed cyst images were rotated to render a cross-section appropriate for measurements. When conducting immunofluorescence analysis on PASE, we have cross-checked HOECHST staining with other antibody staining to ensure that (1) the amniotic ectoderm contains cells that are double positive for both HOECHST and corresponding amniotic marker such as TFAP2A and GATA3; (2) the amniotic ectoderm contains HOECHST positive nuclei that are negative for pluripotency markers such as NANOG and OCT4.

### Live-cell imaging

Live-cell imaging was conducted using the IncuCyte ZOOM live-cell analysis system (Essen Bioscience), with a 20 × objective and at a time interval of 2 h. Time-lapse images and videos were exported with the IncuCyte ZOOM software (version 2015A). Of note, the time-lapse images shown in Figs 4a and 5a were rotated by 180° from the original videos for presentation.

### RNA isolation from fetal human amniotic epithelial cells

Fetal placentae were obtained from the Allegheny Reproductive Health Center under the approval of the University of Pittsburgh’s Internal Review Board and prior written consents of the donors^40^. Human amnion samples were procured with the prior written consent of the donors. Any patients who tested positive for human immunodeficiency virus, hepatitis B virus, hepatitis C virus, tuberculosis, *Chlamydia trachomatis*, *Neisseria gonorrhoeae*, syphilis, or any placenta showing macroscopic abnormalities were excluded. Human amniotic epithelial (AE) cells were enzymatically isolated from the amniotic membrane as previously described^41^. In brief, the amnion layer was mechanically separated from the chorion layer and washed several times PBS without calcium and magnesium to remove blood. To dissociate AE cells, the amniotic membrane was incubated at 37 °C with 0.05% trypsin containing 0.53 mM EDTA-4Na (Life Technologies). The digest from the first 10 min of trypsin digestion was discarded to exclude debris. Cells from the second and third 40 min digests were pooled and washed three times with PBS and immediately cryopreserved with embryonic stem cell-qualified fetal bovine serum containing 10% dimethyl sulfoxide. Total RNA was isolated from the cryopreserved samples by using TRIzol (ThermoFisher) or a DNA-Free RNA Kit (Zymo Research).

### qRT-PCR analysis

Reverse transcription of isolated total RNA was performed using the iScript^TM^ cDNA synthesis Kit (Bio-Rad). qRT-PCR analysis was performed using the Quantitect Sybr Green MasterMix (QIAGEN) and primers listed in Supplementary Table 2 on a CFX Connect^TM^ Real-Time System (Bio-Rad) for 40 cycles. Human *GAPDH* was used as an internal control for quantifying relative gene expression by using the 2^−∆Ct^ method^42^. All analyses were performed with 2–3 technical replicates. RNA extracts from *n* = 3 fetal human amnion samples were used. The investigator conducting the qRT-PCR analyses was blinded as to the group allocation.

### Data availability

The authors declare that all data supporting the findings of this study are available within the article and its supplementary information files or from the corresponding author upon reasonable request.

## Electronic supplementary material

Supplementary Information

Supplementary Movie 1

Supplementary Movie 2

Supplementary Movie 3

Supplementary Movie 4
