# Supplementary material for: A pluripotent stem cell-based model for post-implantation human amniotic sac development
Source: Nat Commun. 2017 Aug 8;8:208. doi: 10.1038/s41467-017-00236-w (PMC5547056; doi:10.1038/s41467-017-00236-w)
Supplement: Supplementary file 1 — Supplementary Information [file 41467_2017_236_MOESM1_ESM.pdf]

File Name: Supplementary Information
Description: Supplementary Figures, Supplementary Tables, Supplementary References.

File Name: Supplementary Movie 1
Description: Representative time-lapse movie showing dynamic morphogenesis during the development of a PASE. Time stamps indicate the total hours of culture. Scale bar, 50 µm. The same cyst is presented in Fig. 4a (with 180° rotation).

File Name: Supplementary Movie 2
Description: Representative time-lapse movie showing the progressive emergence of the EMT and PS-like phenotype in a PASE. Time stamps indicate the total hours of culture. Scale bar, 50 µm. The same cyst is presented in Fig. 5a (with 180° rotation).

File Name: Supplementary Movie 3
Description: Representative time-lapse movie showing the initial development of an unstable asymmetric cyst and its subsequent conversion to a fully squamous cyst. Time stamps indicate the total hours of culture. Scale bar, 50 µm. The same cyst is presented in Fig. 10a.

File Name: Supplementary Movie 4
Description: Representative time-lapse movie showing the development of a squamous cyst through circumferential progressive squamous morphogenesis. Time stamps indicate the total hours of culture. Scale bar, 50 µm. The same cyst is presented in Supplementary Fig. 13a.

Supplementary Figures and Legends
Supplementary Figure 1

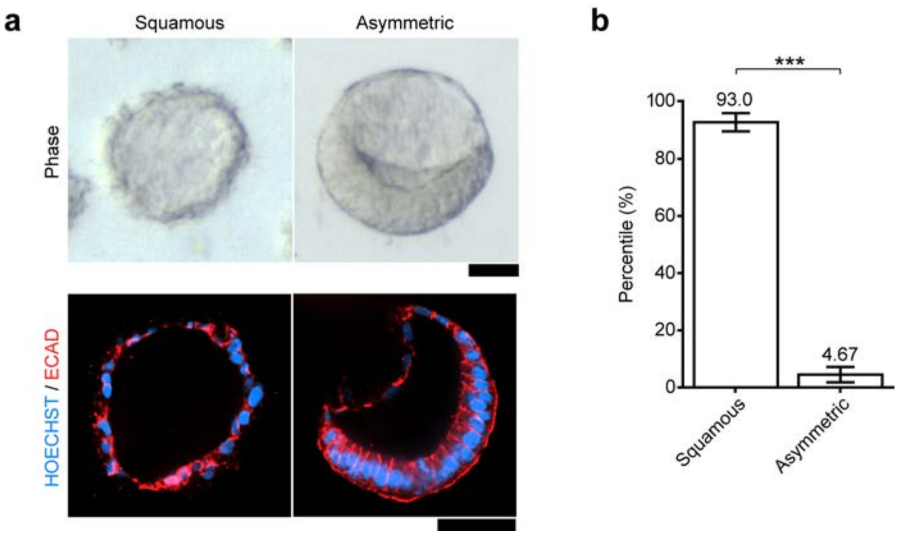

Supplementary Figure 1. hPSC form both squamous and asymmetric cysts in 3D culture. (**a**) Representative phase contrast (top) and confocal immunofluorescence (bottom) micrographs showing a squamous cyst (left) and an asymmetric cyst (right) observed in the 3D culture system. Cysts were stained for ECAD (red). HOECHST (blue) counterstains nuclei. n = 5 independent experiments. Scale bars, 50 µm. (**b**) Bar plot showing percentages of squamous and asymmetric cysts in the 3D culture on day 5. hPSC were plated at 30,000 cells cm^{-2} at the beginning of culture. n = 3,662 and 168 for squamous and asymmetric cysts, respectively, out of a total of 3,948 cysts from n = 8 biological replicates. Data represent the mean ± s.d. P-values were calculated using paired, two-sided Student's t-test. ***: $P < 0.001$. The remaining 118 cysts exhibited a columnar morphology[1]. Asymmetric cyst formation was consistently observed in all experiments (n > 18 independent experiments).

Supplementary Figure 2

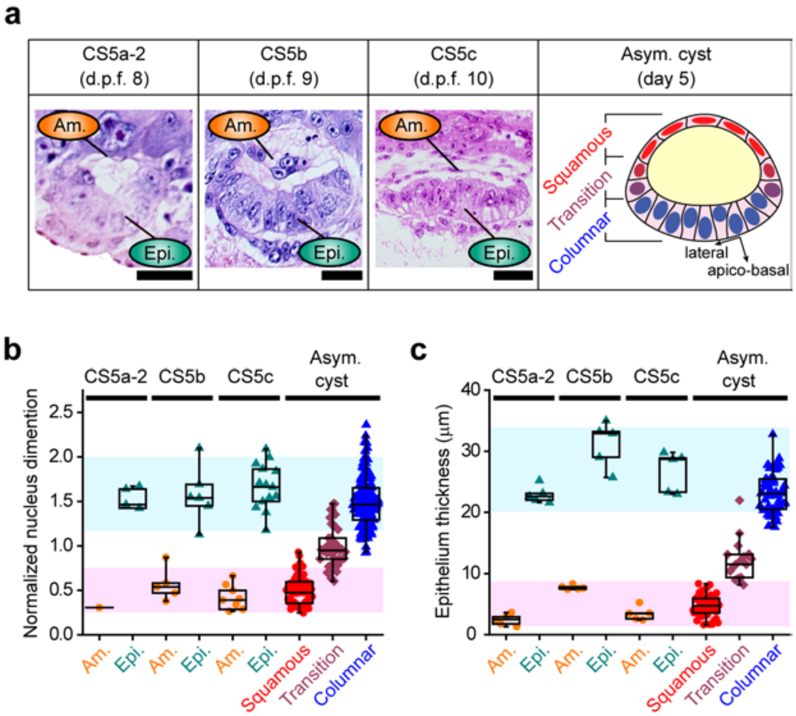

Supplementary Figure 2. Asymmetric cysts morphologically resemble the human amniotic sac. (**a**) Comparison of Carnegie stage embryos to the asymmetric cyst (the embryo sections are the same as those shown in **Fig. 1d**). The cartoon summarizes morphological patterning in the asymmetric cyst. Scale bars, 30 µm. (**b&c**) Tissue/region-specific measurements of normalized nuclear dimension (*apico-basal* : *lateral* dimension of nuclei) (**b**) and epithelium thickness (**c**) in human embryos and in the asymmetric cyst as indicated. Box: 25-75%, bar-in-box: median, and whiskers: 1 and 99%. For embryos measured in **b**, *n* = 1, 5, and 8 prospective/definitive amniotic ectoderm cells (Am.), and *n* = 4, 6, and 14 epiblast cells (Epi.), from CS5a-2, CS5b, and CS5c embryos shown in **a**, respectively, were analyzed. Of note, since the CS5a-2 embryo does not show amniotic ectoderm cells as definitive as CS5b and CS5c embryos do, we called the squamous portion at the roof of the CS5a-2 embryo "prospective amniotic ectoderm" (labeled as "Am."), which appears to enclose the amniotic cavity with the epiblast at the floor. For asymmetric cysts measured in **b**, *n* = 68 squamous, 47 transitional, and 143 columnar cells,

from $n = 12$ cysts, were analyzed. Each dot represents a single cell in **b**. For the CS5a-2, CS5b, and CS5c embryos measured in **c**, 5 measurements were performed for amniotic and epiblast regions, respectively, for each embryo section shown in **a**. For asymmetric cysts measured in **c**, 5 measurements were conducted for each cyst at 5 evenly distributed locations, which are subsequently grouped to specific regions per definition shown in **a**. Each measurement was plotted as a single dot in **c**.

Supplementary Figure 3

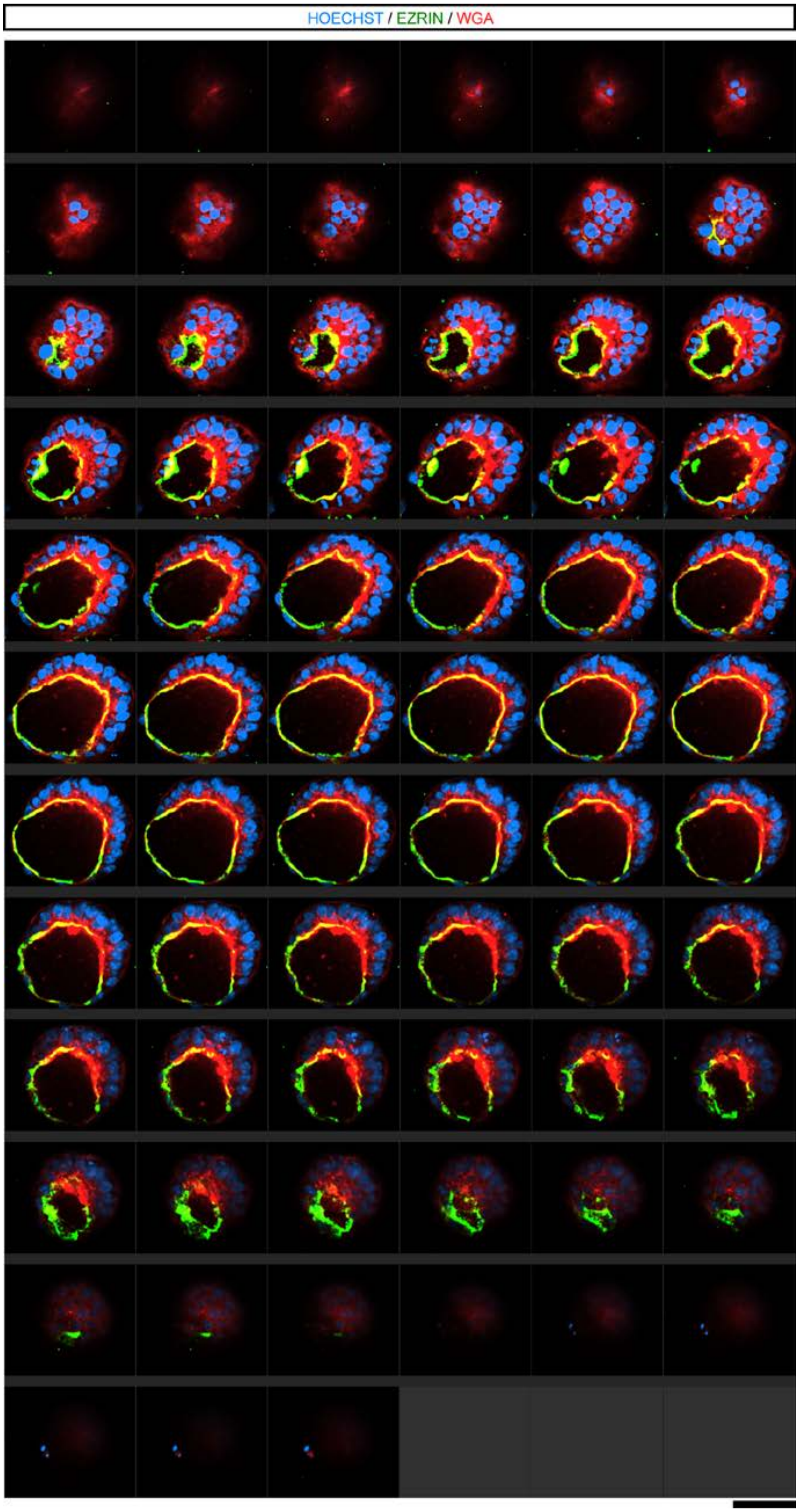

Supplementary Figure 3. Full *Z*-stack confocal micrographs of an asymmetric cyst.

A representative asymmetric cyst on day 5 was stained for EZRIN (green) and WGA (red). A bipolar pattern of cell morphology is visible and a continuous EZRIN+, WGA-enriched single apical lumen that faces inward can be seen throughout the cyst. HOECHST (blue) counterstains nuclei. $n = 5$ independent experiments. Scale bar, 50 µm.

Supplementary Figure 4

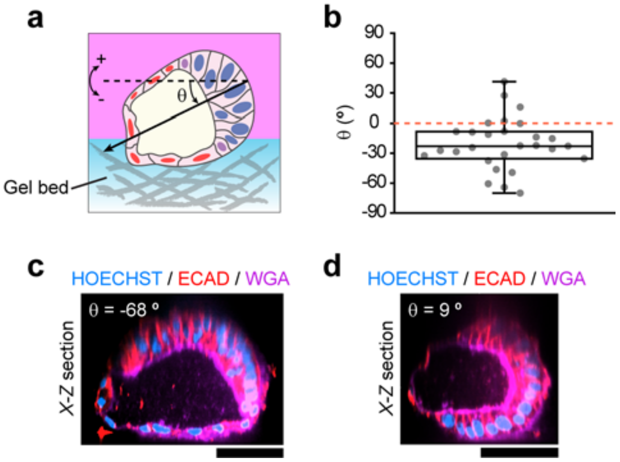

Supplementary Figure 4. Vertical orientation of the asymmetric cyst.

(**a**) Schematic showing the definition of cyst orientation angle θ, which is negative when the squamous side of the cyst is closer to the gel bed. (**b**) Quantitated cyst orientation angle θ from $n = 28$ cysts. Box: 25-75%, bar-in-box: median, and whiskers: 1 and 99%. Baseline of $\theta = 0°$ is drawn (red dashed line) for reference. (**c&d**) *X-Z* confocal sections showing representative cysts stained for ECAD (red) and WGA (purple), with cyst orientation angle $\theta = -68°$ (**c**) and 9° (**d**), respectively. HOECHST counterstains nuclei. $n = 5$ independent experiments. Scale bars, 50 µm.

76 **Supplementary Figure 5**

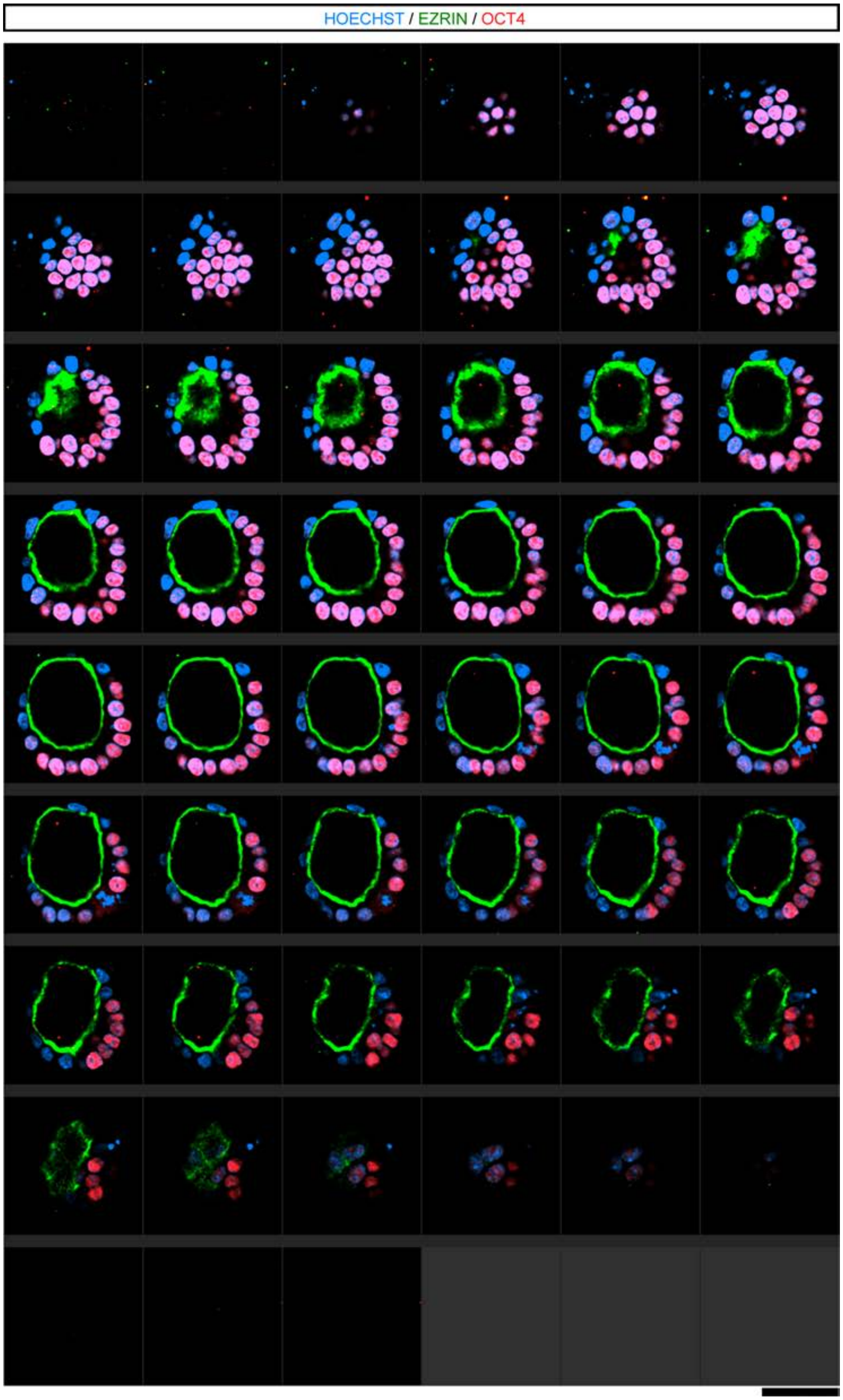

77

78

Supplementary Figure 5. Full *Z*-stack of confocal micrographs showing an embryonic disc-like structure in the asymmetric cyst.

This representative asymmetric cyst (same as shown in **Fig. 3a**) was stained for EZRIN (green) and OCT4 (red). HOECHST (blue) counterstains nuclei. Nuclear staining of OCT4 is only prominent in the thick, columnar side of the cyst, and is lost in the flattened squamous side, consistent with the notion that the columnar side represents an embryonic disc-like structure. Scale bar, 50 µm.

Supplementary Figure 6

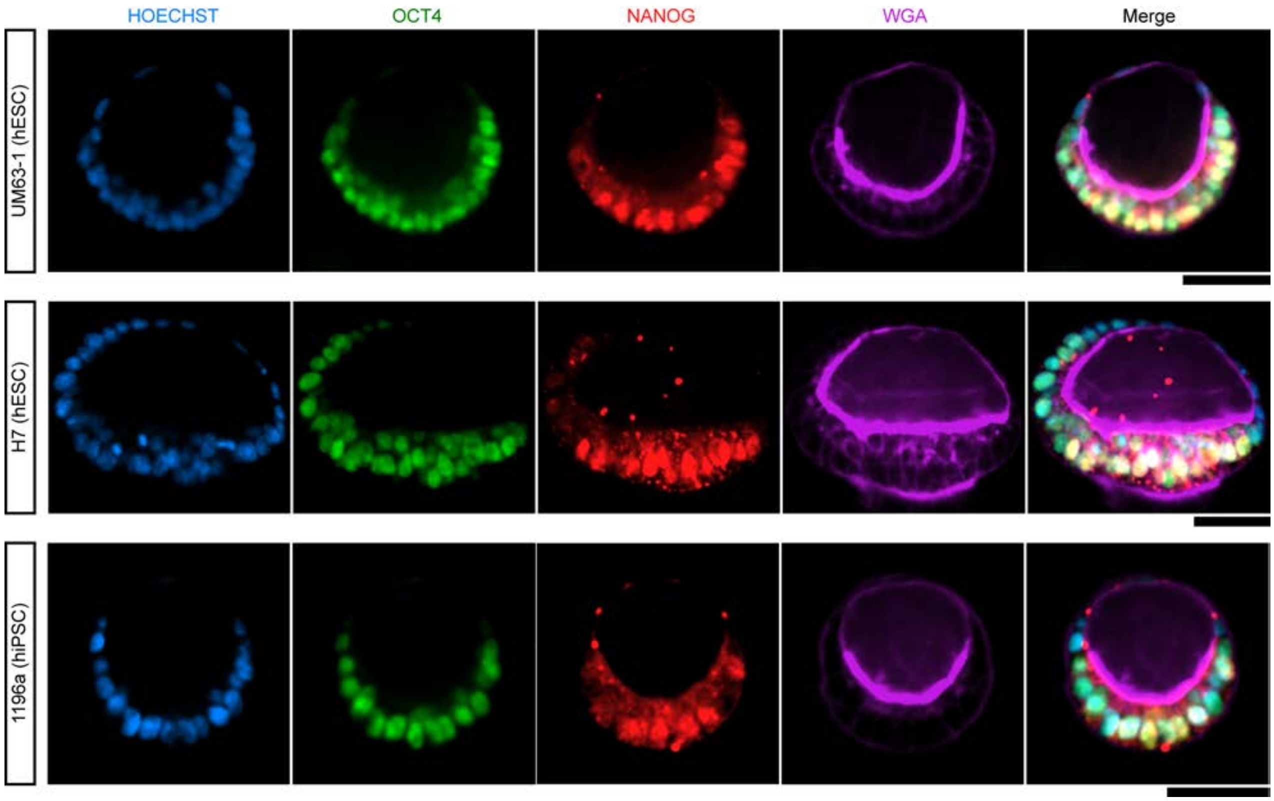

Supplementary Figure 6. Generation of asymmetric cysts using multiple hPSC lines.

Representative confocal micrographs showing asymmetric cysts derived from two hESC lines (UM63-1, top; H7, middle), and one hiPSC line (1196a, bottom) on day 5 as indicated. The cysts were stained for OCT4 (green), NANOG (red) and WGA (purple). HOECHST counterstains nuclei. *n* = 2 independent experiments for each hPSC line. Scale bars, 50 µm.

Supplementary Figure 7

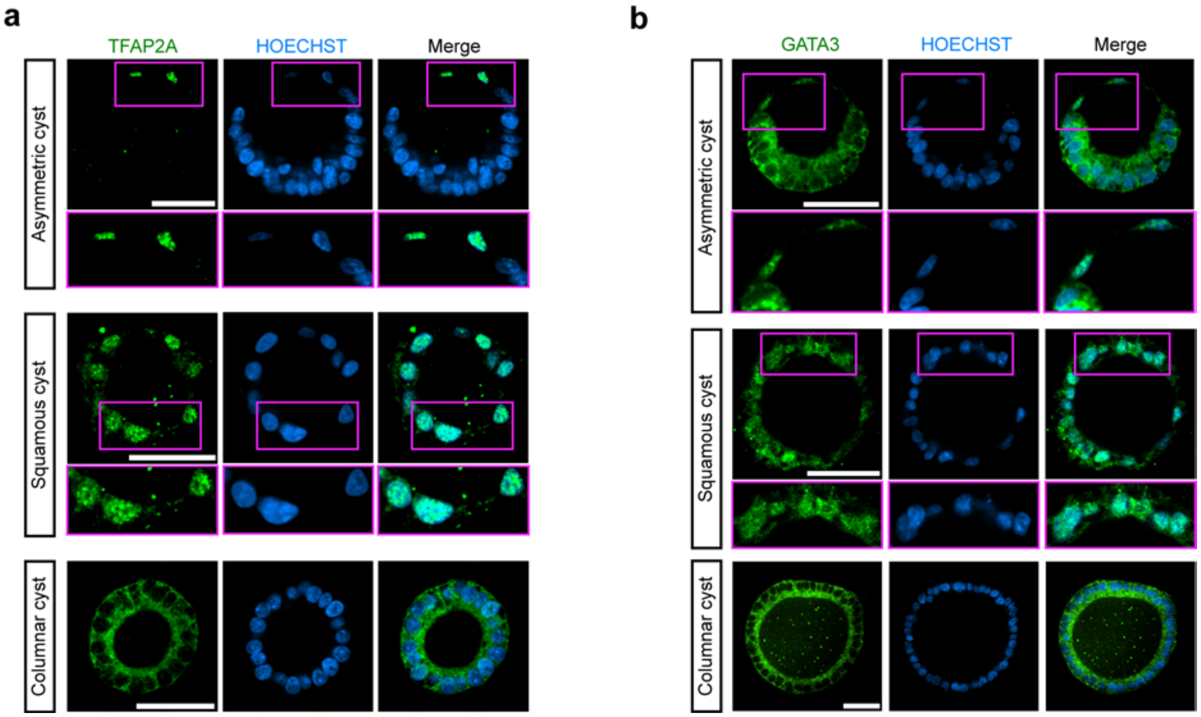

Supplementary Figure 7. Examination of amniotic markers in the squamous cells. (**a**) Representative confocal micrographs showing an asymmetric cyst (top; same as shown in **Fig. 3d**), a squamous cyst (middle), and a columnar cyst (bottom). Cysts were stained for TFAP2A (green). HOECHST (blue) counterstains nuclei. Zoom-in images are shown for the boxed regions at the amniotic pole. The asymmetric cyst and the squamous cyst were generated in the 3D amniogenic culture system. The columnar cyst was generated in Glass-3D culture system[1]. *n* = 3 independent experiments. Scale bars, 50 µm. (**b**) Representative confocal micrographs showing an asymmetric cyst (top; same as shown in **Fig. 3e**), a squamous cyst (middle), and a columnar cyst (bottom). Cysts were stained for GATA3 (green). HOECHST (blue) counterstains nuclei. Zoom-in images are shown for the boxed regions at the amniotic pole. The asymmetric cyst and the squamous cyst were generated in the 3D amniogenic culture system. The columnar cyst was generated in Glass-3D culture system[1]. *n* = 2 independent experiments. Scale bars, 50 µm.

Supplementary Figure 8

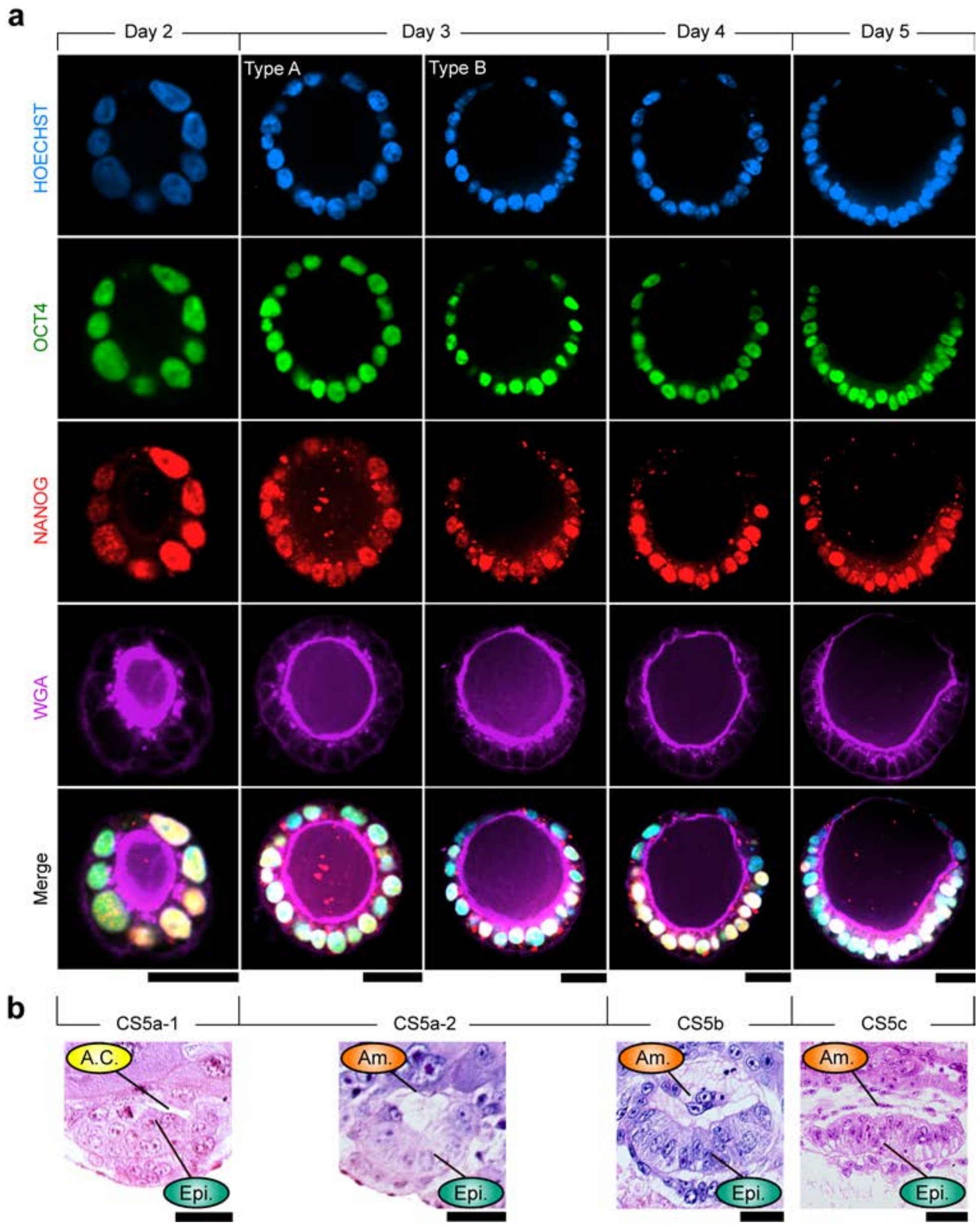

Supplementary Figure 8. Temporal evolution of PASE development.

(**a**) Representative confocal micrographs showing PASE from day 2-5 (as shown in **Fig. 4b**), stained for OCT4 (green), NANOG (red), and WGA (purple). HOECHST (blue) counterstains nuclei. Since nuclear staining of NANOG typically exhibits heterogeneous intensity in cultured hPSC, only cells in which nuclear staining of NANOG is completely absent were considered to have lost NANOG expression. Scale bars, 30 µm. (**b**) Sections of Carnegie stage (CS) 5a-1[2], 5a-2[3], 5b[2], and 5c[4] human embryos at d.p.f.

7, 8, 9, and 12, respectively, which spans from peri- to post-implantation developmental stages. Sections were obtained from the Virtual Human Embryo Project. The sections of CS5a-2, CS5b, and CS5c embryos are the same as shown in **Fig. 1d**. A.C.: pro-amniotic cavity; Am.: (prospective) amniotic ectoderm; Epi.: epiblast. Scale bars, 30 µm.

Supplementary Figure 9

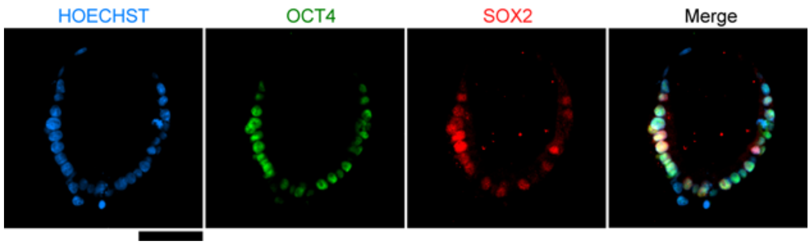

Supplementary Figure 9. SOX2 expression is decreased in disseminating cells. Representative confocal micrographs showing a day 5 PASE that exhibits an EMT phenotype on the columnar side, stained for OCT4 (green) and SOX2 (red). HOECHST (blue) counterstains nuclei. *n* = 3 independent experiments. Scale bar, 50 µm.

Supplementary Figure 10

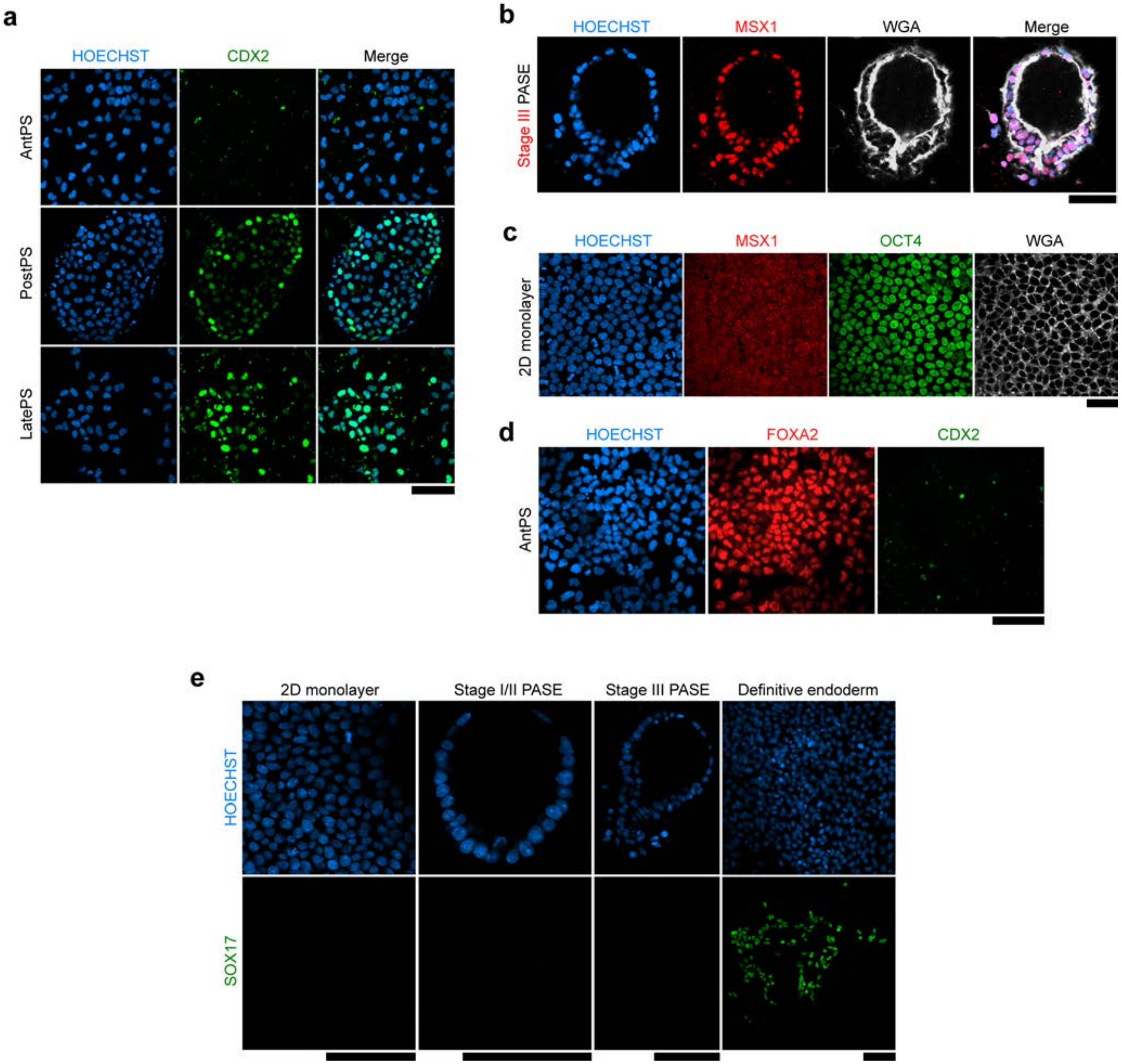

Supplementary Figure 10. Characterization of primitive streak markers in PASE.

(**a**) Representative confocal micrographs showing anterior primitive streak (AntPS; top), posterior primitive streak (PostPS; middle), and late primitive streak (LatePS; bottom) cells derived from hPSC in 2D culture, stained for CDX2 (green). HOECHST (blue) counterstains nuclei. *n* = 2 independent experiments. Scale bar, 100 µm. (**b**) Representative confocal micrographs showing a stage III stained for

MSX1 (red). WGA (white) co-staining shows cell membrane. HOECHST (blue) counterstains nuclei. *n* = 2 independent experiments. Scale bar, 50 µm. (**c**) Representative confocal micrographs showing undifferentiated hPSC stained for MSX1 (red) and OCT4 (green) under standard 2D monolayer culture, where MSX1 is known to be not expressed[1]. WGA (white) co-staining shows the cell membrane. HOECHST (blue) counterstains nuclei. *n* = 2 independent experiments. Scale bar, 50 µm. (**d**) Representative confocal micrographs showing AntPS cells, stained for FOXA2 (red), and CDX2 (green). HOECHST (blue) counterstains nuclei. *n* = 2 independent experiments. Scale bar, 100 µm. (**e**) Representative confocal micrographs showing undifferentiated 2D hPSC monolayer, stage I/II PASE, stage III PASE, and hPSC-derived definitive endoderm cells, respectively, stained for SOX17 (green). HOECHST (blue) counterstains nuclei. hPSC-derived definitive endoderm cells, which are known to express SOX17, were derived by following an established protocol[5]. *n* = 2 independent experiments. Scale bar, 100 µm.

Supplementary Figure 11

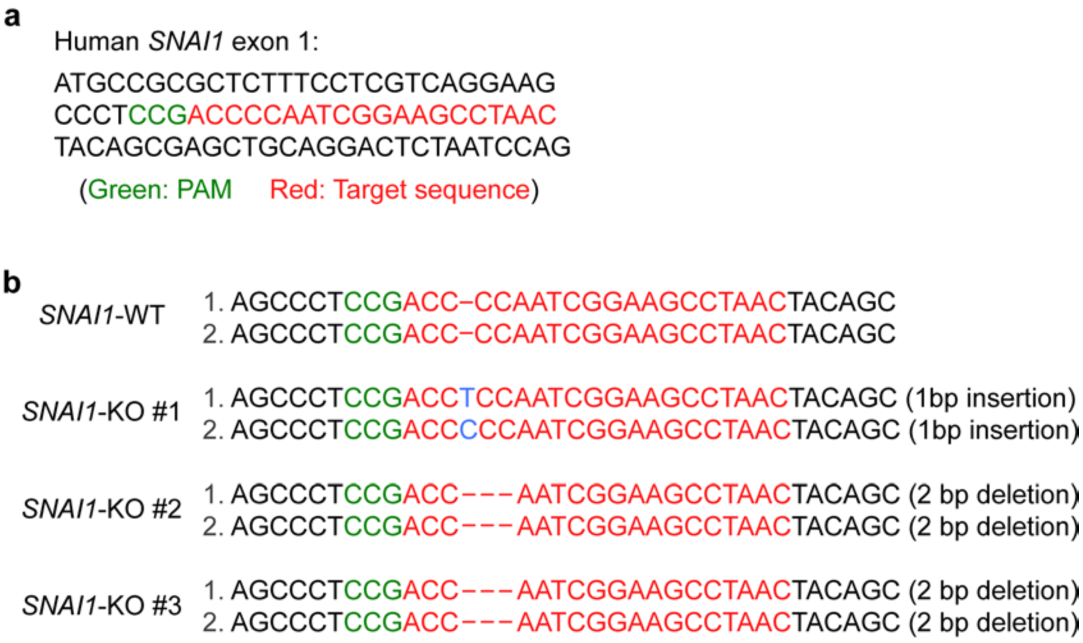

Supplementary Figure 11. Design and validation of *SNAI1*-KO.

(**a**) Sequence of exon 1 in human *SNAI1*. The PAM sequence recognized by the guide RNA (gRNA) is highlighted in green. The 20 base-pair (bp) target sequence is highlighted in red. (**b**) Sequencing of *SNAI1* wild type (*SNAI1*-WT) and three separate *SNAI1*-KO lines, showing frame-shift mutations caused by 1 bp insertion (#1) and 2 bp deletion (#2 and #3).

Supplementary Figure 12

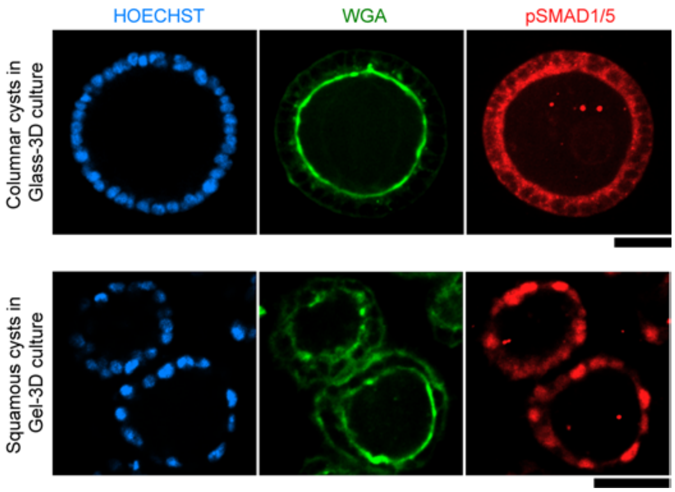

Supplementary Figure 12. Control assay to ascertain pSMAD1/5 antibody specificity. Representative confocal micrographs showing columnar pluripotent cysts[1] formed in Glass-3D (upper) and squamous amniotic cysts[1] formed in Gel-3D (lower) on day 5, stained for pSMAD1/5 (red). WGA (green) stains cell membrane. HOECHST (blue) counterstains nuclei. n = 2 independent experiments. Scale bars, 50 µm.

Supplementary Figure 13

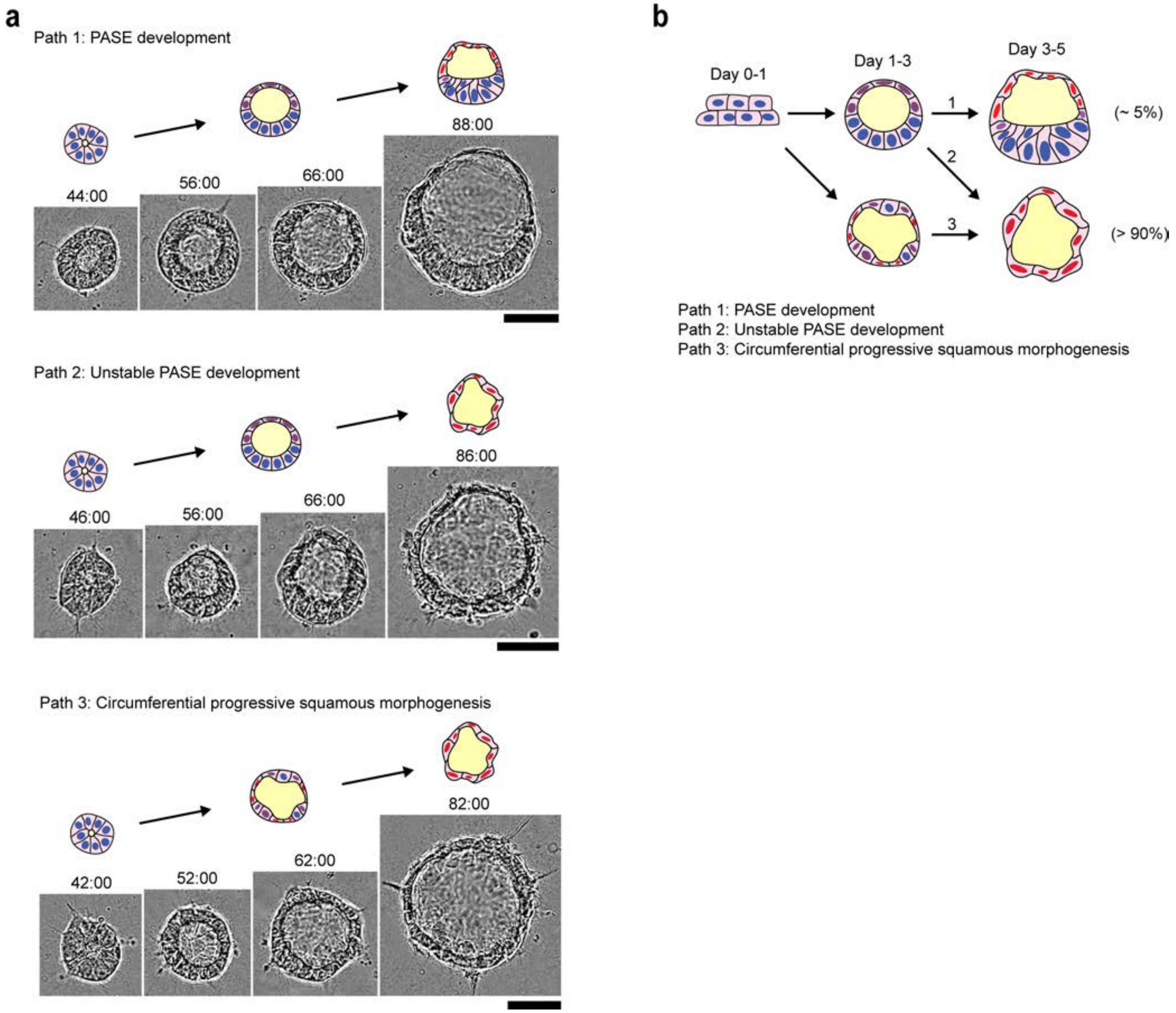

Supplementary Figure 13. Distinct developmental pathways for PASE vs. squamous tissues. (**a**) Representative time-lapse sequences showing stable PASE development (Path 1; same sequence as shown in **Fig. 4a**); unstable PASE development (Path 2; same sequence as shown in **Fig. 10a**); and circumferential progressive squamous morphogenesis (Path 3; also see **Supplementary Movie 4**). Path 1 leads to stable PASE formation while Paths 2&3 lead to fully squamous amniotic ectoderm-like cyst formation. Of note, all three pathways start from uniformly columnar cysts. $n = 3$ independent experiments. Scale bars, 50 μm. (**b**) Schematic summarizing the developmental pathways towards PASE

vs. squamous amniotic ectoderm-like cysts in the current system.

Supplementary Tables

Supplementary Table 1. List of primary antibodies.

Protein	Species	Application	Catalog No.	Vendor
**E-CADHERIN**	Mouse	1:500 (ICC)	610181	BD Biosciences
**E-CADHERIN**	Rabbit	1:100 (ICC)	ab15148	Abcam
**β-CATENIN**	Mouse	1:200 (ICC)	610153	BD Biosciences
**EZRIN**	Mouse	1:2000 (ICC)	E8897	Sigma-Aldrich
**OCT4**	Mouse	1:200 (ICC)	SC-5279	Santa-Cruz Biotechnology
**OCT4**	Rabbit	1:500 (ICC)	2750	Cell Signaling Technology
**NANOG**	Rabbit	1:500 (ICC)	4903S	Cell Signaling Technology
**SOX2**	Rabbit	1:1000 (ICC)	09-0024	Stemgent
**TFAP2A**	Mouse	1:100 (ICC)	3B5	Developmental Studies Hybridoma Bank
**GATA3**	Mouse	1:100 (ICC)	SC-268	Santa-Cruz Biotechnology
**BRACHYURY**	Rabbit	1:100 (ICC)	SC-20109	Santa-Cruz Biotechnology
**CDX2**	Mouse	1:500 (ICC)	MU392AUC	Biogenex
**FOXA2**	Rabbit	1:500 (ICC)	WRAB-1200	Seven Hills Bioreagents
**pSMAD1/5**	Rabbit	1:100 (ICC)	9516S	Cell Signaling Technology
**MSX1**	Rabbit	1:500 (ICC)	NBP2-30052	Novus Biologicals
**SOX17**	Goat	1:500 (ICC)	AF1924	R&D Systems

Supplementary Table 2. List of qRT-PCR primers.

Gene	Primer Sequences (5' -> 3')	Reference
SOX2	*Forward:* GCTTAGCCTCGTCGATGAAC	*NA*
	Reverse: AACCCCAAGATGCACAACTC	*NA*
GAPDH	*Forward:* CTCTGCTCCTCCTGTTCGAC	*NA*
	Reverse: TTAAAAGCAGCCCTGGTGAC	*NA*
GATA3	*Forward:* GCCCCTCATTAAGCCCAAG	PrimerBank[6]
	Reverse: TTGTGGTGGTCTGACAGTTCG	PrimerBank
TFAP2A	*Forward:* GCATATCCGTTCACGCCGAT	Tadeu *et al.*[7]
	Reverse: GGGAGATTGACCTACAGTGCC	Tadeu *et al.*[7]

NA: not applicable.

Supplementary References

1. Shao, Y., *et al.* Self-organized amniogenesis by human pluripotent stem cells in a biomimetic implantation-like niche. *Nature Mater.* **16**, 419-425 (2017).

2. Hertig, A. T. and Rock, J. Two human ova of the pre-villous stage, having a development age of about eight and nine days respectively. *Contributions to Embryology* Carnegie Institution of Washington, **33**, (1945).

3. Hertig, A. T. and Rock, J. Two human ova of the pre-villous stage, having a development age of about seven and nine days respectively. *Contributions to Embryology* Carnegie Institution of Washington, **31**, (1949).

4. Hertig, A. T. and Rock, J. Two human ova of the pre-villous stage, having an ovulation age of about eleven and twelve days respectively. *Contributions to Embryology* Carnegie Institution of Washington, **29**, (1941).

5. McCracken, K. W., Howell, J. C., Wells, J. M. and Spence, J. R. Generating human intestinal tissue from pluripotent stem cells in vitro. *Nat. Protoc.* **6**, 1920-1928 (2011).

6. Wang, X. W., Spandidos, A., Wang, H. J. and Seed, B. PrimerBank: A PCR primer database for quantitative gene expression analysis, 2012 update. *Nucleic. Acids. Res.* **40**, D1144-D1149 (2012).

7. Tadeu, A. M. B., *et al.* Transcriptional profiling of ectoderm specification to keratinocyte fate in human embryonic stem cells. *PLoS One* **10**, e0122493 (2015).
